# Population genomics of *Fusarium graminearum* reveals signatures of divergent evolution within a major cereal pathogen

**DOI:** 10.1371/journal.pone.0194616

**Published:** 2018-03-27

**Authors:** Amy C. Kelly, Todd J. Ward

**Affiliations:** United States Department of Agriculture, Agricultural Research Service, Peoria, Illinois, United States of America; Soonchunhyang University, REPUBLIC OF KOREA

## Abstract

The cereal pathogen *Fusarium graminearum* is the primary cause of *Fusarium* head blight (FHB) and a significant threat to food safety and crop production. To elucidate population structure and identify genomic targets of selection within major FHB pathogen populations in North America we sequenced the genomes of 60 diverse *F*. *graminearum* isolates. We also assembled the first pan-genome for *F*. *graminearum* to clarify population-level differences in gene content potentially contributing to pathogen diversity. Bayesian and phylogenomic analyses revealed genetic structure associated with isolates that produce the novel NX-2 mycotoxin, suggesting a North American population that has remained genetically distinct from other endemic and introduced cereal-infecting populations. Genome scans uncovered distinct signatures of selection within populations, focused in high diversity, frequently recombining regions. These patterns suggested selection for genomic divergence at the trichothecene toxin gene cluster and thirteen additional regions containing genes potentially involved in pathogen specialization. Gene content differences further distinguished populations, in that 121 genes showed population-specific patterns of conservation. Genes that differentiated populations had predicted functions related to pathogenesis, secondary metabolism and antagonistic interactions, though a subset had unique roles in temperature and light sensitivity. Our results indicated that *F*. *graminearum* populations are distinguished by dozens of genes with signatures of selection and an array of dispensable accessory genes, suggesting that FHB pathogen populations may be equipped with different traits to exploit the agroecosystem. These findings provide insights into the evolutionary processes and genomic features contributing to population divergence in plant pathogens, and highlight candidate genes for future functional studies of pathogen specialization across evolutionarily and ecologically diverse fungi.

## Introduction

*Fusarium graminearum* is the primary cause of Fusarium head blight (FHB), a devastating disease affecting wheat, barley and other cereal crops worldwide [1,2]. During host infection, the fungus destroys grain and produces toxic metabolites, like deoxynivalenol, that contaminate cereals and damage the immune, gastrointestinal and reproductive systems of humans and animals [1,3]. Repeated FHB outbreaks in major cereal-producing regions around the world have resulted in substantial reductions in cereal yield, quality and value, and billions of dollars in economic losses [4,5].

In North America FHB has re-emerged in recent decades, with outbreaks occurring regularly across major cereal growing regions [2,6,7]. North America has historically been dominated by a single, genetically diverse population of FHB pathogens (referred to as NA1) that typically produce the 15-acetyl-deoxynivalenol (15ADON) trichothecene toxin analog [8]. However, a highly virulent, introduced population of *F*. *graminearum* (referred to as NA2) that typically produce 3-acetyl-deoxynivalenol (3ADON) has rapidly increased in frequency in some regions compared to the endemic population [9–12]. Although the geographic origin of NA2 remains uncertain, this population exhibits reduced diversity consistent with a clonal expansion following a bottleneck, which likely occurred after founder isolates were transcontinentally introduced [11]. Trichothecenes are known virulence factors in wheat [13], and chemotype differences may therefore be contributing to the invasion of NA2/3ADON isolates in some regions. However, NA2 isolates can also deposit more toxin in grain, grow faster and spread more quickly than NA1 isolates on certain lines of wheat [11,12,14,15], indicating that other fitness traits may be involved in pathogen adaptive shifts. Changes in FHB pathogen composition therefore warrants concern about food safety and the sustainability of FHB-resistant crops and fungicides.

Additional diversity in the *F*. *graminearum* gene pool was recently discovered in the Midwestern US, where a novel group of isolates that have the 3ADON haplotype at the trichothecene biosynthetic gene cluster (*TRI-*cluster), but produce a distinct trichothecene analog, 3α-acetoxy, 7α,15-dihydroxy-12,13-epoxytrichothec-9-ene (NX-2) were found to cause FHB on wheat [16–18]. The evolution of NX-2 in *F*. *graminearum* likely reflects a change in selective constraint on Tri1, a trichothecene-modifying P450 enzyme located on chromosome 1, outside of the *TRI-*cluster [16]. Recent inquiries into the demographic history of NX-2 *F*. *graminearum* were unable to resolve their relationship with NA1 and NA2; genotypes based on tandem repeat markers suggested these isolates share the NA1 genetic background [19], whereas restriction site polymorphisms indicated shared ancestry with NA2 [20]. However, both of these previous studies failed to reject the hypothesis that the NX-2 chemotype may be associated with a distinct population.

The significant changes in FHB pathogen diversity in North America have resulted in geographic clines in chemotype frequency [11] and region-specific biases in FHB population composition; NA2 is dominant in parts of the Midwestern US and western and Maritime provinces of Canada, NA1 is dominant in eastern Canadian provinces of Québec and Ontario [19], and NX-2 *F*. *graminearum* are exclusively found in the northern US and southern Canada [16,21]. The geographic distribution and population dynamics of these pathogens suggest that FHB populations inhabiting North America have been influenced by a complex selective landscape [19], and may be differentially adapted across their continental range. Currently, however, we know very little about the genetic variation in *F*. *graminearum* populations that could be contributing to phenotypic divergence in FHB pathogens.

Genome comparisons of two *F*. *graminearum* isolates highlighted genomic regions with high evolutionary potential [22], and strain-specific genes that may influence virulence [23]. However, genomic diversity across the broad spectrum of FHB pathogens in North America has never been evaluated. Therefore, to identify candidate genes for functional studies of FHB pathogen diversity, we sequenced the genomes of 60 *F*. *graminearum* comprising NA1, NA2 and NX-2 isolates sampled across North America, and 11 genomes representing four related *Fusarium* outgroup species (S1 Table). We performed phylogenomic and Bayesian analyses to gain a genome-wide perspective on the population identity of NX-2 isolates and clarify their genetic relationships with endemic NA1 and introduced NA2 pathogens. Then, we scanned the genome for signatures of population divergence, by searching for localized reductions in nucleotide diversity and pronounced inter-population differentiation that occurs around alleles targeted by selection [24,25]. In addition, we assembled the first *F*. *graminearum* pan-genome to identify gene sets that distinguished populations and may therefore contribute to phenotypic variation in FHB pathogens. Biological functions of the candidate selected genes and gene sets that differentiated populations were then investigated to make inferences about fungal divergence, pathogen population dynamics and future prospects for crop disease management.

## Results

### Population genomic structure

We generated whole-genome sequences for 60 isolates of *F*. *graminearum*, 10 isolates from three related species in the *Fusarium graminearum* species complex (FGSC), *F*. *gerlachii* (*N =* 3), *F*. *louisianense* (*N =* 2), and *F*. *boothii* (*N =* 5), and an isolate of *F*. *pseudograminearum*, a member of the *Fusarium sambucinum* species complex that is an outgroup to the FGSC [26]. On average, genome re-sequencing of the *F*. *graminearum* isolates resulted in 50X coverage across 99% of the PH-1 reference genome. The AT rich (>90% AT) regions near the centromeres and ends of chromosomes [27] were generally not well resolved due to repetitive DNA content, and were therefore excluded from further analyses. A total of 505,748 quality-filtered, nuclear SNPs identified across the 60 isolates of *F*. *graminearum* were retained for population genomic analyses, which comprised a subset of the 1,083,417 SNPs identified across all isolates and species in the FGSC. The genomic distribution of SNPs within *F*. *graminearum* varied extensively, consistent with previous reports demonstrating that regions near subtelomeres and central portions of chromosomes 1, 2 and 4, corresponding to putative ancestral chromosome fusion sites, had the highest density of polymorphic sites [22,23,28].

**Fig 1:**
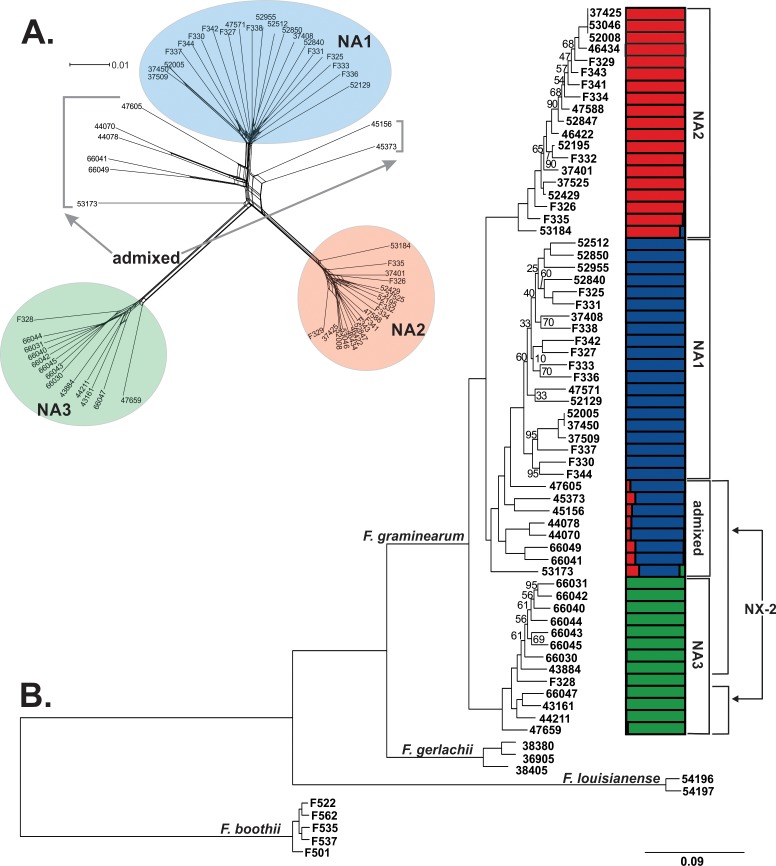
Evolutionary history and population structure of North American *F*. *graminearum*. A phylogenetic network (A) and maximum likelihood phylogeny (B) was inferred from SNPs identified by reference-based mapping of whole genome sequences to the PH-1 *F*. *graminearum* genome (Ensembl, version RR). Taxa comprised 20 NA1/15ADON isolates, 20 NA2/3ADON isolates, 20 NX-2 isolates, and for the maximum likelihood analyses, representatives from three FGSC species were included: *F*. *boothii* (*N =* 5), *F*. *gerlachii* (*N =* 3) and *F*. *louisianense* (*N =* 2). Different colors indicate clustering assignment of each isolate in the three populations inferred from Bayesian analyses: NA2 = red, NA1 = blue and NA3 = green. A. SplitsTree4 [98] was used to construct a network wherein *F*. *graminearum* isolates are represented by terminal nodes, and relationships are depicted as branches with parallel edges indicating reticulate events (i.e. recombination, gene transfer). B. In the maximum likelihood phylogeny, all bootstrap values were 100%, except those indicated at branch nodes. The tree was rooted with *F*. *boothii* and drawn to scale, with branch lengths measured in the number of substitutions per site. Colored bars indicate Bayesian estimates of ancestry (*q*) for each isolate in the three populations.

Preliminary Bayesian clustering analyses indicated that sampling SNPs at 5 kb intervals across the genome provided sufficient resolution to assess population structure and admixture, while significantly reducing the computational load and minimizing the influence of linkage. Analyses of this reduced dataset comprising 6,852 variant sites provided strong evidence for three distinct populations, which was in agreement with evolutionary relationships depicted by a reticulating phylogenetic network of *F*. *graminearum* that accounted for intraspecific recombination (Fig 1A). Across three replicate Bayesian clustering analyses, likelihood and Δ*K* values were maximized for models with *K =* 3. Population structure inferred from the optimal model indicated that endemic NA1/15ADON and introduced NA2/3ADON *F*. *graminearum* belonged to two separate populations, based on *q-*value estimates of each isolate’s ancestry in each population (average *q*_*NA1*_ *=* 1.0, average *q*_*NA2*_ *=* 0.99). However, a single 3ADON isolate (F328) and the majority (60%) of NX-2 *F*. *graminearum* were assigned to a third genetic population, hereafter referred to as NA3 (average *q*_*NA3*_ = 1.0). The remaining eight NX-2 isolates had admixed genomes, in that they shared the majority of their ancestry with the NA1 population (average *q*_*NA1*_ *=* 0.85), but were assigned with significantly lower probability compared to other isolates (*P <* 0.001) and also shared a proportion of ancestry with the NA2 (average *q*_*NA2*_ *=* 0.13) and NA3 (average *q*_*NA3*_ *=* 0.02) populations.

The maximum likelihood phylogeny inferred from all 1,083,417 SNPs identified in isolates of *F*. *graminearum* and FGSC species was consistent with Bayesian clustering analyses and phylogenetic networks of *F*. *graminearum*, in that the three genetic populations comprised strongly supported (100% of bootstraps) clades (Fig 1B). The phylogeny indicated that the NA3 clade split from the common ancestor of the NA1 and NA2 populations following divergence of *F*. *graminearum* from *F*. *gerlachii* and *F*. *louisianense*, two sister species found in the US.

Collectively, Bayesian and phylogenomic analyses indicated that eight isolates with the NX-2 toxin type clustered with NA1/15ADON isolates. Recombination events between admixed NX-2 *F*. *graminearum* and NA1, NA2 and NA3 *F*. *graminearum* were evident in the phylogenetic network as well (Fig 1A). Moreover, in the maximum likelihood phylogeny (Fig 1B), admixed isolates occupied a basal position within a clade that included all of the NA1/15ADON isolates, consistent with expectations for isolates with a recombinant background. A separate maximum likelihood phylogeny of *TRI1*, the gene responsible for differences between NX-2 and 3ADON or 15ADON toxin types, confirmed that the gene sequences encoding the NX-2 trichothecene toxin had a single evolutionary origin and did not arise separately in admixed and NA3 isolates (S1 Fig), consistent with previous studies [16]. Further, genome-wide averages of sliding-window *F*_*ST*_ values indicated significantly higher levels of gene flow between admixed isolates and NA1 isolates (average *F*_*ST*_ = 0.16, *P <* 0.001), and to a lesser extent, between admixed isolates and NA2 isolates (average *F*_*ST*_ = 0.43, *P <* 0.001) compared to all other population pairs. As such, the NX-2 *F*. *graminearum* associated with the NA1 population appear to have genomes that reflect a history of recombination between populations. Therefore, to limit the confounding effects of gene flow, these admixed NX-2 isolates were excluded from further population-based analyses, but were examined separately where appropriate.

### Genome-wide diversity of *F*. *graminearum* populations

Genomic scans of diversity performed in non-overlapping, 10 kb sliding windows revealed distinct patterns of DNA variation within NA1, NA2 and NA3. Average nucleotide diversity (*π*) and polymorphism (*θ*) indicated reduced genetic variation in NA2, and a relatively low number of nucleotide differences between isolates (Table 1). Furthermore, Tajima’s *D* values, which describe the spectrum of allele frequencies in each population, were significantly higher and the variance of *D* values was significantly larger (Bartlett’s test *P <* 0.001) in NA2 than in the other two populations. These genetic patterns are consistent with a recent bottleneck in NA2. In contrast, the endemic NA1 population was the most diverse, and isolates were genetically heterogeneous as indicated by the relatively large number of pairwise nucleotide differences (Table 1), and the positive average Tajima’s *D* [29]. NA3 showed slightly lower levels of diversity than NA1, but values of Tajima’s *D* were relatively low and negatively biased, which could indicate a recent population expansion [30]. The demographic histories inferred from the observed allele frequency spectrums of the three populations were consistent with the evolutionary history of North American *F*. *graminearum* reported previously [11,16,19,20], however, average Tajima’s *D* values suggested deviations from neutral expectations, and should thus be interpreted with caution.

**Table 1:** Average genomic diversity within *F*. *graminearum* populations.

Population	θ^1^	*π*	Tajima's *D*
NA1	0.0012^a^	0.0013^a^	0.14^b^
NA2	0.0006^c^	0.0006^c^	0.15^a^
NA3	0.001^b^	0.001^b^	-0.34^c^

^1^ nucleotide polymorphism per site based on the number of segregating sites.

^a-c^ The genomic distributions of each sliding-window summary statistic were compared across populations using nonparametric tests. Different letters indicate significant (*P <* 0.001) differences in the population distributions.

### Signatures of selection highlight genomic regions involved in population divergence

**Fig 2:**
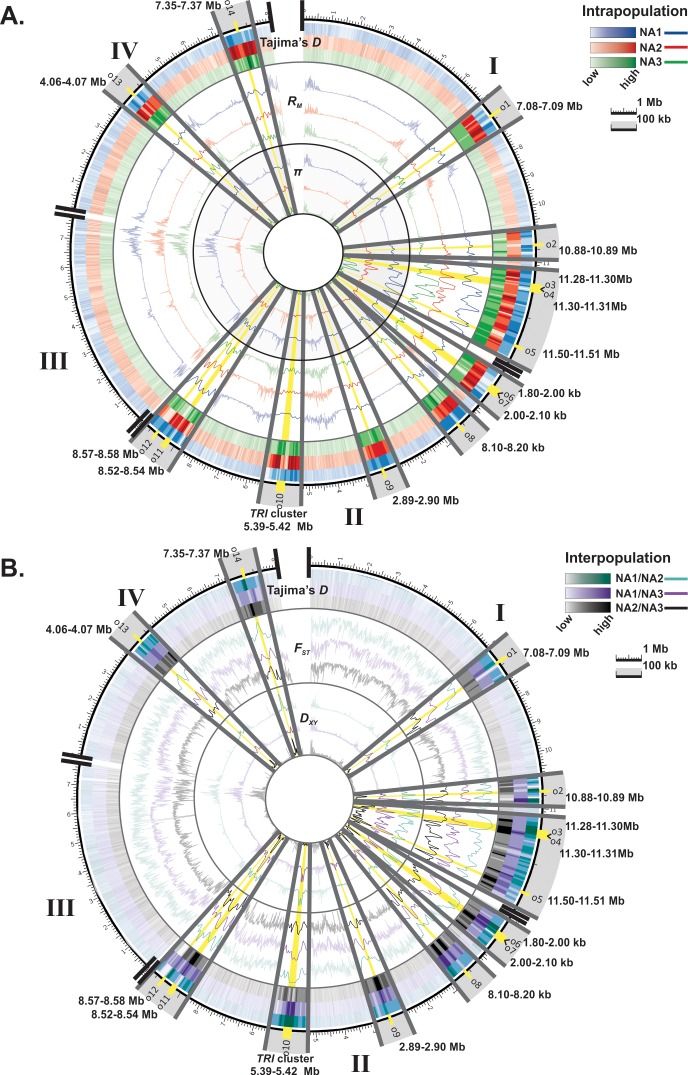
Genomic distribution of outlier regions exhibiting signatures of selection. Sliding-window values of intrapopulation diversity and recombination (Panel A, Tajima’s *D*, *π* and *R*_*m*_) and interpopulation differentiation (Panel B, Tajima’s *D*, *D*_*xy*,_ and *F*_*ST*_) were calculated in 10 kb windows to identify genomic regions with signatures of selection in NA1 (omitting admixed NX-2 isolates), NA2 or NA3 populations of *F*. *graminearum*. The fourteen outliers (o1-o14, yellow highlight) showed significant (*P* < 0.001) divergence between populations based on pairwise (interpopulation) values of Tajima’s *D*, *D*_*xy*,_ and *F*_*ST*_, coupled with significantly reduced diversity (*π*) within the divergent population(s). Significance was assessed by comparing observed sliding-window values of each metric against a null genome-wide distribution derived through random permutation.

After characterizing genome-wide patterns of diversity within populations, we utilized combined signals from several sliding-window statistics to pinpoint genomic regions contributing to divergence in the three populations. We identified 14 regions with genetic signatures of selection (i.e., outliers, Table 2, Fig 2). These regions exhibited significant interpopulation divergence (*P <* 0.001 for *D*_*xy*_, *F*_*ST*_ and Tajima’s *D* between population pairs) and reduced diversity (*P <* 0.001 for *π*) within one or more populations as compared to a null distribution of values generated through random permutation. Patterns of genetic variation in the vicinity of these regions indicated that ten of the 14 outliers had signatures of selection that localized to a single 10 kb window, while the other four had signatures that extended across consecutive 10 kb windows, with three outliers spanning 20 kb and one spanning 30 kb (Table 2). All but one of the outliers (o9, chromosome 2) were clustered in the subtelomeric and interstitial portions of chromosomes (Fig 2), within known polymorphic islands [22,28].

**Table 2:** Genomic regions showing genetic signatures of selection in *F*. *graminearum* populations.

Outlier region^1^	Significant window (chromosome: coordinates)	Genes (InterPro domains)^2^		References
			Targeted population(s)^3^	
o1	1:7,080,001–7,090,000	FGRRES_15944 (Zinc finger transcription factor), FGRRES_02168 (aminotransferase), FGRRES_02169 (peptidase), FGRRES_02170 (glycoside hydrolase/ chitinase), FGRRES_02171 (glutathione-dependent formaldehyde-activating enzyme), FGRRES_02172 (α,β hydrolase fold)	NA2	
o2	1:10,880,001–10,890,000	FGRRES_17361 (Major facilitator superfamily), FGRRES_10437, FGRRES_17362 (pleckstrin, CDC24 calponin), FGRRES_13758 (Ankyrin repeat)	NA2, NA3 (*P<*0.05)	
o3	1:11,280,001–11,290,000	FGRRES_10552, FGRRES_10553, FGRRES_20102, FGRRES_10554 (secreted cysteine-rich effector), FGRRES_10555, FGRRES_10556	NA2	[31]
	1:11,290,001–11,300,000	FGRRES_17388_M, FGRRES_10557_M, FGRRES_10558 (Heterokaryon incompatibility), FGRRES_10559	NA2	
o4	1:11,300,001–11,310,000	FGRRES_10560, FGRRES_10561 (Hce2 effector protein, Glycoside hydrolase/chitinase, LysM), FGRRES_10562	NA3^Fg^	[32]
o5	1:11,500,001–11,510,000	FGRRES_17407 (Urease, Amidohydrolase), FGRRES_17408 (Zinc finger transcription factor), FGRRES_17409	NA3 ^Fb, Fg, Fl^	
o6	2:180,001–190,000	FGRRES_13457 (Zinc finger transcription factor), FGRRES_08017 (citrate lyase domain), FGRRES_13456 (dehydrogenase), FGRRES_13455 (Zinc finger transcription factor), FGRRES_17114 (General substrate transporter)	NA1, NA3 ^Fb, Fg^	
	2:190,001–200,000	FGRRES_17113 (Gluconolactonase), FGRRES_08021, FGRRES_08022 (Peptidase), FGRRES_08023 (Cytochrome P450)	NA1, NA3 ^Fg^	
o7	2:200,001–210,000	FGRRES_08024 (Glucose dehydrogenase), FGRRES_08025 (secreted Cyanovirin-N), FGRRES_08026, FGRRES_13453	[NA1,NA2], NA3 (*P<*0.01) ^Fg^	[31]
o8	2:810,001–820,000	FGRRES_08248 (hydrolase), FGRRES_17074_M (decarboxylase), FGRRES_08250, FGRRES_08251 (Gluconolactonase)	[NA1,NA2], NA3(*P<*0.05) ^Fl^	
o9	2:2,890,001–2,900,000	FGRRES_08850 (SH3 domain), FGRRES_08851 (methylcitrate dehydratase), FGRRES_16955 (DNA photolyase, Cryptochrome)	NA3	[33]
o10	2:5,390,001–5,400,000	*TRI8*, *TRI7*, *TRI3*,	NA1^Fb^, [NA2,NA3]	[34,35]
	2:5,400,001–5,410,000	*TRI4*, *TRI6*, *TRI5*, *TRI10*, *TRI9*	NA1^Fb^, [NA2, NA3 (*P =* 0.06)]	
	2:5,410,001–5,420,000	*TRI11*, *TRI12*, *TRI13*, *TRI14*	NA1^Fb^, [NA2, NA3]	
o11	2:8,520,001–8,530,000	FGRRES_04599 (Glucose dehydrogenase), FGRRES_04600, FGRRES_15251 (secreted cysteine-rich effector), FGRRES_12220, FGRRES_16086 (Protein kinase-like)	NA3^Fb, Fl^	[31]
	2:8,530,001–8,540,000	FGRRES_12218, FGRRES_12217 (ankyrin repeat), FGRRES_04603 (Alpha carbonic anhydrase), FGRRES_04604 (Cullin), FGRRES_04605 (Glucose dehydrogenase)	[NA1^Fg, Fl^, NA2 ^Fg, Fl^], NA3 (*P<*0.05)	
o12	2:8,570,001–8,580,000	FGRRES_12216, FGRRES_12215 (HeLo, heterokaryon, incompatibility), FGRRES_04620 (α,β hydrolase fold), FGRRES_12214 (secreted cysteine-rich effector), FGRRES_15258	NA2, NA3 ^Fb, Fl^	[31]
o13	4:4,060,001–4,070,000	FGRRES_07631 (General substrate transporter), FGRRES_07632 (Major facilitator superfamily), FGRRES_07633, FGRRES_07634 (Sodium symporter)	NA1(*P<*0.01)^Fb^, NA2 ^Fg, Fl^	
o14	4:7,360,001–7,370,000	*PGL1* (PKS3, PP-binding domain), FGRRES_13527 (*SNF2*-related DEAD helicase), FGRRES_09181 (carboxylesterase, plant effector), FGRRES_09180 (UV radiation resistance protein)	NA2	[36,37]

^1^Outliers (o1-o14) showed significantly (*P* < 0.001, unless otherwise indicated) elevated divergence between populations based on pairwise (interpopulation) values of Tajima’s *D*, *D*_*xy*,_ and *F*_*ST*_, and significantly reduced diversity (*π*) within the targeted population(s), based on null distributions generated through random permutation.

^2^Genes and domains are based on annotations in the PH-1 *Fusarium graminearum* genome [27] (Ensembl, version RR).

^3^Populations with the same allele are grouped by brackets, and superscripts indicate the allele was also found in outgroup species: Fg = *F*. *gerlachii*, Fl = *F*. *louisianense*, Fb = *F*. *boothii*.

**Fig 3:**
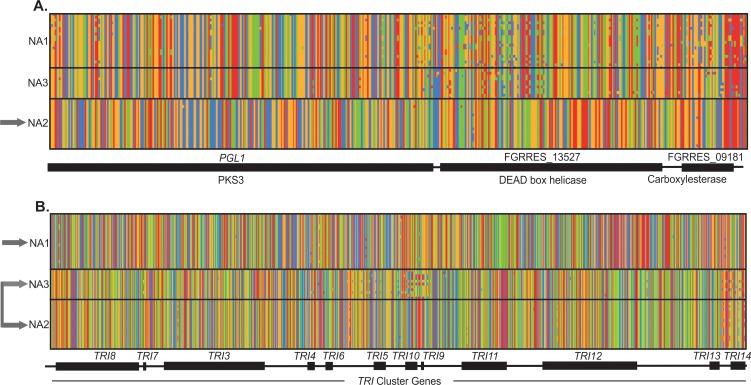
SNP haplotype alignments show divergence of NA1, NA2 and NA3 *F*. *graminearum* at outlier regions. For each alignment, rows correspond to the polymorphisms found in a single isolate and columns show the SNPs found at each variant site. Gene designations and conserved domains are based on the PH-1 *F*. *graminearum* genome [27] (Ensembl, version RR). Panels A-B show examples of the genetic patterns observed at outliers. A. chromosome 4: 7,360,000–7,367,500; a single population (NA2, grey arrow) exhibited reduced within-population diversity and high divergence from the other two populations. B. chromosome 2: 5,390,000–5,420,000, *TRI* cluster genes; diversity was reduced in all three populations and two highly divergent haplotypes (grey arrows: 15ADON haplotype in NA1 and 3ADON haplotype in NA2/NA3) were segregating among the three populations.

For six outliers evidence of selection was limited to haplotypes from a single population, i.e., only one of the three populations exhibited reductions in diversity and high divergence from the other two populations (Table 2, Fig 3A), suggesting that a single selective sweep had targeted one population. In contrast, at the remaining eight regions multiple, population-specific outlier haplotypes were observed, such that two or more populations exhibited reductions in diversity and high divergence from the other populations (Fig 3B), suggesting that soft sweeps had resulted in the fixation of multiple, divergent alleles in populations, or that the same genomic regions had experienced recurrent selective events. At all of these regions, sequences within the outlier population(s) were highly similar, but had an extremely large number of fixed differences compared to sequences in the other populations (*D*_*xy*_ *P <* 0.001). Although outliers tended to reside in genomic regions that generally exhibited frequent recombination and are known to harbor recombination hot-spots [22,38], in most cases, there was a notable reduction in the number of cross-over events within 10 kb outlier windows of the affected population(s) as compared to the flanking regions (Fig 2).

Admixed isolates had sequences that were 98–100% identical to sequences of NA1, NA2 or NA3 isolates at all outlier regions. Further, for nine of the 14 outliers, sequences in at least some of the *F*. *graminearum* isolates (Table 2) were found in related FGSC species as well. Phylogenies of genes residing within outlier regions were discordant with the genome-wide phylogeny based on SNPs (Fig 1B), in that trees constraining *F*. *graminearum* isolates to a monophyletic clade were significantly (*P <* 0.001) worse than maximum likelihood inferred phylogenies, with a single exception (FGRRES_02171). The majority of outliers (11 of 14) contained at least one gene that showed trans-specific patterns of inheritance, in that alleles from one *F*. *graminearum* population were more closely related to the alleles in other FGSC species than to the alleles in different *F*. *graminearum* populations (S2 Fig). For example, NA1 and *F*. *boothii* isolates shared the 15ADON haplotype at the *TRI-*cluster genes located on chromosome 2, while all NA2, NA3 and admixed isolates with the NX-2 chemotype shared the 3ADON *TRI-*cluster haplotype (Table 2). Outlier regions did not distort our whole-genome phylogeny, however, as the topology of the genome-wide SNP phylogeny did not change when these 14 regions were omitted (S3 Fig).

**Fig 4:**
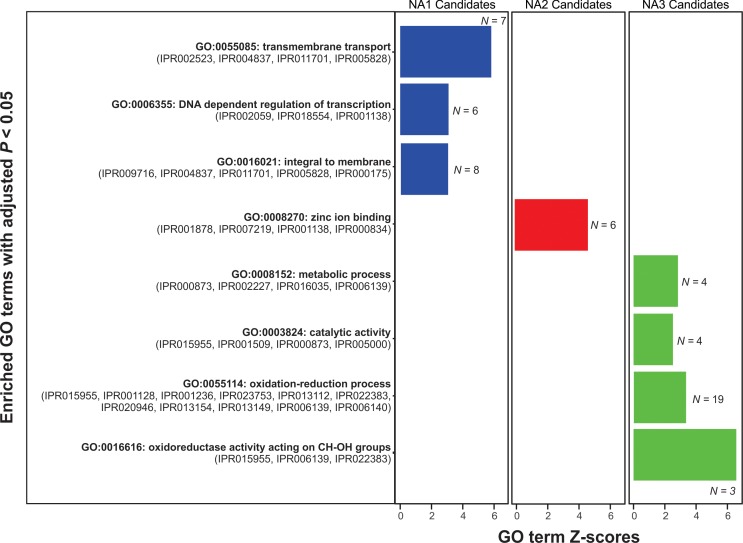
*F*. *graminearum* populations are differentiated by divergent gene sets that are enriched for different functions. Functional enrichment was assessed based on Gene Ontology (GO) terms assigned to domains annotated in candidate genes identified in NA1 (left, blue bars), NA2 (center, red bars) and NA3 (right, green bars). Candidate genes included genes in outlier regions showing signatures of selection and differentially conserved genes in each population. Hypergeometric tests were performed by calculating Z-scores that compared the relative frequency of GO terms assigned to each candidate gene set to GO terms assigned to all functionally annotated *F*. *graminearum* genes. Z-scores and the number of domains in each gene set (*N*) are only shown for GO terms that were significantly overrepresented after a Benjamini–Hochberg adjustment for multiple testing (adjusted *P* < 0.01) [134]. InterPro domains associated with each GO term are listed in parentheses below the term description in bold type.

Of the 81 genes residing in outlier regions (Table 2), 56 contained domains that were functionally annotated in the InterPro database. Functional enrichment analyses of domains associated with a Gene Ontology (GO) term (*N =* 31) indicated that zinc ion binding domains (GO:0008270), specifically, fungal transcription factors (IPR007219, IPR001138), and membrane proteins (GO:0016021; IPR011701, IPR005828, IPR000175) were significantly overrepresented among the population differentiating genes in outlier regions as compared to all *F*. *graminearum* genes (Fig 4). Additionally, a substantial proportion of outliers (71%) contained genes that encoded one or more hydrolytic or degradative enzymes, and at least four contained genes with predicted functions related to pathogenicity, including short, cysteine-rich, secreted effector proteins [31]. In addition, another outlier locus encoded a secreted carboxylesterase (FGRRES_09181) that is likely involved in the breakdown of plant material during FHB infection [36,39], flanked by a DEAD helicase (FGRRES_13527) and a polyketide synthase (Pgl1) (Fig 3A). The strongest signals of selection were observed across the length of the *TRI-*cluster genes (Table 2, Fig 2, Fig 3B) encoding enzymes and proteins that are directly involved in the biosynthesis of trichothecene mycotoxins.

Not all effector genes identified in outliers were implicated in pathogenicity. Two of these regions contained secreted, cysteine-rich effector proteins that colocalized with fungal incompatibility cell death domains (HeLo, PF14479; HET, PF06985; [40]). Two other outliers encoded secreted, antimicrobial proteins (Table 2). Moreover, all three populations were highly differentiated at the putative anti-fungal protein FGRRES_10561, containing an Hce2 toxin effector domain (PF14856, homolog of Ecp2 effector) fused with a chitinase domain (PF00704, glycoside hydrolase 18) [32]. Gene phylogenies and protein alignments indicated that distinct variants of this toxin were present in NA1, NA2 and NA3 (S2 Fig).

### Heterogeneity in gene content in FHB pathogen populations

After mapping reads to the PH-1 genome sequence, we performed a *de novo* assembly of the unmapped reads from each isolate to explore genomic features that were not represented in the reference genome. This allowed us to assemble a pan-genome for North American *F*. *graminearum*, comprising core genes shared by all isolates and accessory genes that were missing in one or more isolates. On average, orphan contig assembly resulted in an additional 705 kb of nucleotide sequence per isolate, though the percentage of AT bases in these sequences was higher than the genome-wide average; that is, the AT content was 56% for orphan contigs versus 52% in the reference genome. This suggests that these sequences tended to reside close to the AT-rich regions near centromeres and the ends of chromosomes [27]. Due to their low sequence complexity, most orphan contigs could not be anchored to specific chromosomal regions using bioinformatics approaches. However, Sanger sequencing into the flanking regions of a subset of orphan contigs (*N =* 5) placed them in the telomere proximal regions of chromosomes 1 and 3.

**Fig 5:**
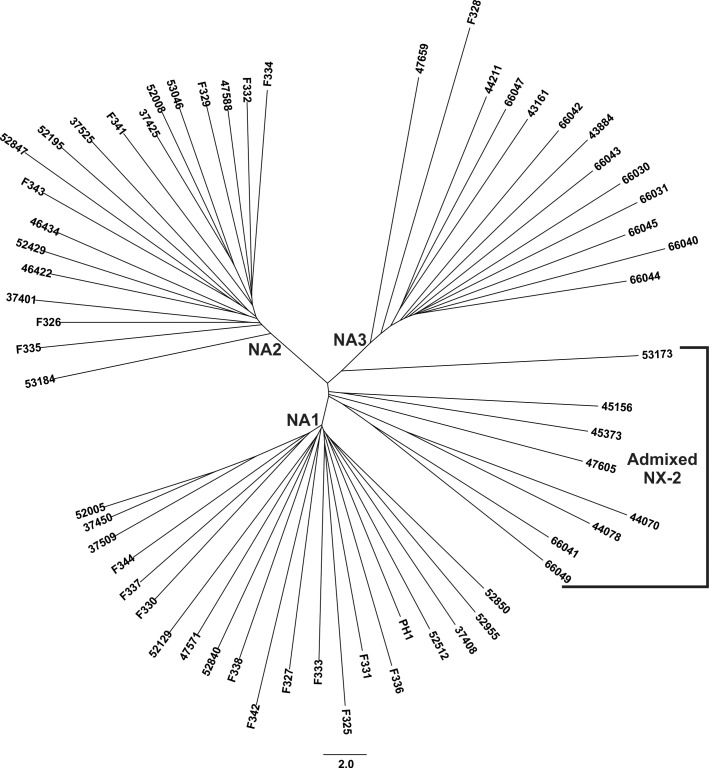
Dendrogram of the *F*. *graminearum* pan-genome. Pairwise distances were computed between genomes based on gene presence/absence polymorphism, and the resulting matrix was used to cluster isolates with the neighbor joining method implemented in MEGA 7 [126]. Branch lengths represent the number of genes that differ in presence/absence status between strains, such that genomes with identical gene content would have a distance of 0.

To determine if differences in gene content contributed to population divergence, we examined presence/absence polymorphism in homologs of all genes present in the reference genome and genes present on orphan contigs in the 60 sampled genomes. We utilized stringent criteria for ortholog assignment (single linkage clustering with inclusion threshold of *D*_*i*,*j*_ ≥ 0.5) that were able to distinguish 97% of all annotated reference genes. A total of 13,632 genes were present in all 60 genomes and the PH-1 reference genome, though a proportion of these (9%) contained premature stop codons or were missing start codons in a subset of genomes and were thus classified as separate orthologs. Still, core genome size may be slightly overestimated, as our clustering algorithm based on BLAST-alignment scores was unlikely to distinguish full-length orthologs from proteins variants with short, carboxy terminal truncations in some cases. Nonetheless, 1,681 genes were considered accessory genes because they were not conserved among all genomes examined. That is, orthologs were missing in one or more genomes, or exhibited considerable divergence from the reference sequence (< 62% identity on average) such that BLAST-based clustering partitioned them into a distinct ortholog group. Accessory genes were relatively rare among sampled isolates, and 27% were unique to a single isolate (S4 Fig), suggesting that many of these genes are poorly conserved or were recently acquired in the sampled isolates. Locations of accessory genes that could be mapped to the PH-1 reference sequence (*N =* 512), overlapped extensively with high diversity, frequently recombining regions of the genome near subtelomeric and interstitial portions of chromosomes [22,27]. Functional enrichment analyses of GO terms assigned to the 1,681 accessory genes indicated that zinc ion binding transcription factors (GO:0008270; IPR001878, IPR003604, IPR007219, IPR000679, IPR001138, IPR006026), protein binding domains such as WD40, ankyrin and tetratricopeptide repeats (GO:0005515; IPR001810, IPR001680, IPR001214, IPR002110, IPR019734, IPR003877), and nuclear associated DNA binding proteins (GO:0003676, GO:0005634; IPR001878, IPR011129, IPR003604, IPR013087, IPR007219, IPR001138, IPR004367) were significantly overrepresented (*P <* 0.01) in the accessory genome as compared to all *F*. *graminearum* genes. A dendrogram based on gene presence/absence polymorphisms in core and accessory genes from the 60 isolates was highly consistent with the maximum likelihood phylogeny and population assignments based on SNP diversity (Fig 5). Isolates from the same genetic population shared similar gene sets, with admixed isolates being primarily associated with the NA1 cluster, and to a lesser extent, the NA3 cluster. A few isolates, however, showed considerable genomic divergence due to a relatively large number of unique genes. Isolate F328, for example, had 103 genes that were not found in any other *F*. *graminearum* isolates, but most of these (81%) showed significant homology (BLAST *E* < 10^−10^) to proteins from fusaria that are not members of the FGSC (e.g., *F*. *oxysporum*, *F*. *langsethiae*, *F*. *poae*). To confirm the presence of these accessory genes in F328, the fungus was re-isolated from a single conidium, and the resulting isolate was subjected to genome sequence analysis. The accessory gene content of the resulting genome sequence was the same as in the original F328 genome sequence. This finding indicates that the accessory genes in F328 could reflect gene acquisition from other fusaria.

**Fig 6:**
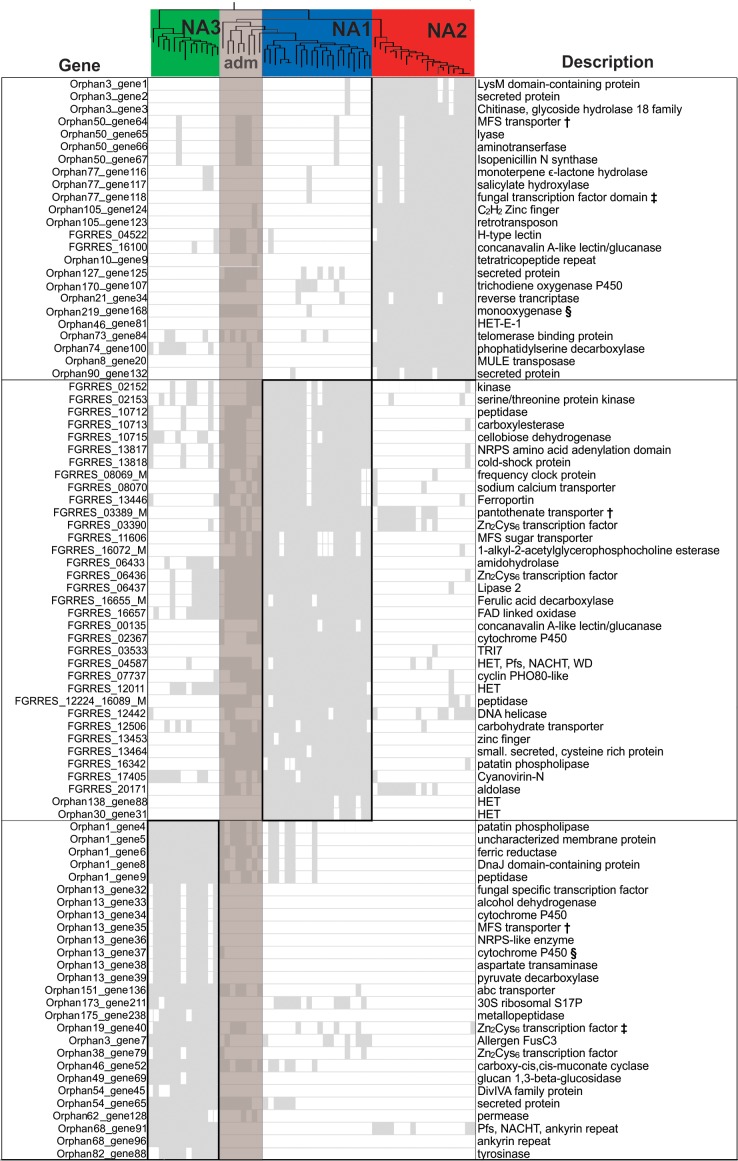
Descriptions of genes that were differentially conserved among populations of *F*. *graminearum*. The matrix indicates the presence (solid grey) and absence (white) of genes that were differentially conserved (*E-*test *P-*value < 0.01) in NA1, NA2 or NA3. Each column in the matrix represents a single isolate and each row represents a gene ortholog. Population structure based on Bayesian clustering (indicated by red, blue and green highlight) is shown, along with the maximum likelihood phylogeny inferred from SNPs. Admixed isolates (adm, shaded brown) were not included in calculations but are shown to emphasize their mosaic genomes. Functional descriptions of the domains encoded by each gene are based on annotations of the PH-1 *F*. *graminearum* genome [27] (Ensembl, version RR) and HMMER, BLAST, BLAST2GO, CD-Search, InterPro and Signal P analyses. Bold symbols (**§**, **‡**, **†**) indicate accessory proteins with similar domain content and structure that were found in different genomic contexts in each population. Differentially conserved genes with uncharacterized functions were not depicted in the figure (*N* = 34).

Population-specific patterns of gene conservation were evident in a small proportion of the accessory genome. Of the 1,681 accessory genes analyzed, 121 genes were differentially conserved among populations (*E* test *P* < 0.01), showing high conservation in one population, but were absent or rare in the other two populations (S2 Table and Fig 6). Only 38 of these genes were exclusive to a single population, in that they were found in most members from one population, but were completely absent in the other two populations; 9 such genes were detected in NA1, 11 in NA2 and 18 in NA3. However, HMMER pattern searches indicated that three of the 121 differentially conserved genes had the same Pfam domain content and order; thus, while BLAST-clustering assigned them to distinct ortholog groups, these proteins may have divergent homologs segregating in different populations (symbols in Fig 6). Nonetheless, these three potential orthologs were found in different genomic contexts and were not present in all isolates, suggesting they are accessory proteins that have diverged in a population-specific manner.

Of the 121 genes that were differentially conserved across populations, 88 could be functionally characterized based on annotations in the PH-1 reference genome, InterPro domains or homology to known proteins (Fig 6). Each population was associated with distinct heterokaryon incompatibility genes (IPR010730, IPR024983). Moreover, degradative enzymes, and in particular hydrolases (IPR013094, IPR016035, IPR015955), but also pathogenicity-related lipases, proteases and effectors [31,36] were commonly found among genes that were differentially conserved. As such, each population had a unique collection of pathogenicity-related genes (Fig 6, S2 Table). Furthermore, we identified numerous putative secondary metabolite biosynthetic genes that were associated with a single population. These genes encoded nonribosomal peptide synthetase (NRPS) enzymes, cytochrome P450 enzymes and a variety of transcription factors and transmembrane transporters (S2 Table). In fact, in each population we identified at least one differentially conserved putative secondary metabolite gene cluster, wherein synthetase enzymes, transporters, transcription factors and modifying enzymes colocalized to the same contig (S2 Table, gene clusters F, I, J and U). Furthermore, functional enrichment analyses of the subset of differentially conserved genes that were assigned GO terms (*N =* 68) showed that transcription regulators (GO:0006355) and domains involved in redox reactions (GO:0016491, GO:0055114, GO:0050660) and transmembrane transport (GO:0055085) were significantly overrepresented (adjusted *P <* 0.01) in these genes.

Taken together, genome scans and gene content surveys showed that each population could be distinguished by a subset of genomic features with unique biological functions. For instance, regions that were differentially conserved or showed signatures of selection in NA1 were enriched for membrane proteins, transmembrane transporters and domains involved in DNA dependent regulation of transcription (adjusted *P <* 0.001, Fig 4). The NA2 population, on the other hand, showed enrichment for zinc finger transcription factors (adjusted *P <* 0.001), whereas the NA3 population had an overabundance of candidate genes with functions related to oxidation-reduction (adjusted *P* < 0.001).

## Discussion

### *F*. *graminearum* population genomics

Our phylogenomic analyses revealed a previously unidentified population of pathogens, herein termed NA3, corresponding to a clade of *F*. *graminearum* that produce the NX-2 toxin. Studies utilizing VNTR and RFLP-based genotyping were unable to elucidate the genetic identity of NX-2 *F*. *graminearum*, but the restricted distribution of these isolates in the northern US and southern Canada suggested they were likely endemic to North America [16,17]. Accordingly, we found no evidence of a recent introduction in NA3, but rather, the relatively high levels of diversity in this population supported an endemic origin and a possible recent expansion (Table 1). Still, despite evidence of gene flow between NA1 and NA3, NA3 isolates have remained genetically distinct (Fig 1) and have genes that distinguish them from isolates belonging to the two predominant populations of FHB pathogens in North America (Fig 5). One possible explanation for these findings is that NA3 isolates recently switched from non-agricultural grasses to domesticated cereals [16,18]. Lofgren et al. (2017) reported that *F*. *graminearum* inhabit diverse wild grass species as endophytes, and wild grasses may be the ancestral hosts for FHB pathogens [41]. Thus, the genomic traits that differentiate *F*. *graminearum* populations may to some degree reflect different evolutionary trajectories taken by these populations as they transitioned from an endophytic lifestyle on wild grasses to pathogens of cultivated cereal.

Indeed, *F*. *graminearum* isolates co-occupying geographic ranges in southern Canada and the northern US are highly substructured compared to pathogens from other regions of the world, which may indicate that they have not coexisted for sufficient time to be in mutation-selection-drift equilibrium. Recent surveys of *F*. *graminearum* conducted in Germany for instance, reported that 213 randomly sampled *F*. *graminearum* over a > 500 km transect belonged to a single, recombining metapopulation [42]. In contrast, our results support the hypothesis that North America harbors multiple *F*. *graminearum* populations that have acquired the ability to infect cultivated cereals brought to the region in the last few hundred years [41].

### Genomic signatures of population-specific selection

After elucidating the rich evolutionary history of North American *F*. *graminearum*, we sought to identify genes underlying pathogen specialization in this species. The outlier regions identified tended to be located near subtelomeres and sites of putative chromosome fusion events (Table 2, Fig 2), wherein patterns of nucleotide variation suggested that selection had contributed to population divergence. At seven of the 14 outlier regions, signatures of selection were detected in multiple populations. For instance, one of the outliers located on chromosome 2 (Table 2, o6) showed patterns consistent with divergent selection targeting NA1 and NA3, and this region contained a gene harboring a SNP associated with a known quantitative trait involved in transcriptional regulation of fungicide resistance in *F*. *graminearum* (FGRRES_13455, SNP:09492 [43]). In addition, the majority of outliers (11 of 14) contained at least one gene that showed evidence of trans-species polymorphism (S2 Fig). Alleles at these outliers may have been recently exchanged among species, or they may be under balancing selection to maintain diversity. The *TRI-*cluster genes, for example, showed patterns of trans-species evolution, and were previously reported to have evolved under balancing selection acting on chemotype polymorphisms within the B trichothecene producing clade of *Fusarium*, which includes all of the FGSC species [15].

Individual populations also showed evidence of being uniquely targeted by positive selection, as five outliers had patterns of variation consistent with an independent sweep of distinct genes/mutations in a single population (Table 2, Fig 3A). Notably, a signature of positive selection was identified in the NA3 population on chromosome 2 between 2.89–2.90 Mb, a region that is directly downstream and approximately 5 kb from a SNP associated with a known quantitative trait that regulates trichothecene production in *F*. *graminearum* [SNP:12335 43], suggesting that this genomic region may encode adaptations that regulate mycotoxin-associated virulence toward sensitive hosts. Likewise, in other fungi similar population-specific sweeps of alleles have been reported at genes encoding adaptive traits [44–46]. In *Neurospora crassa* for example, genomic signatures of population divergence localized to genes that proved to be essential for cold adaptation [44]. In *Microbotryum* fungi many genes with footprints of selection were differentially expressed *in planta*, suggesting that these loci correspond to putative adaptations responding to host-mediated positive selection [46].

Collectively, our genome scans suggested that recurrent episodes of selection in *F*. *graminearum* have allowed independently evolving populations to accumulate divergent alleles at dozens of genes located in rapidly evolving regions of the genome. Core genes encoding membrane proteins, transcription factors and degradative enzymes were preferentially targeted, perhaps indicating that these genes represent essential, but highly dynamic traits that provide evolutionary flexibility to the fungus. However, populations also maintained distinct sets of accessory genes (Fig 6, S2 Table). Comparative genomic studies in bacterial species have shown that such population-specific patterns of gene conservation can result from selection for traits involved in niche specificity [47,48], consistent with the tendency for accessory genes to localize in genomic regions implicated in pathogen specialization [22,23,27,38]. Many of the population-differentiating accessory genes in *F*. *graminearum* had functions related to transmembrane transport, transcription regulation and secondary metabolism suggesting they may also be important for pathogen specialization, though functional studies are warranted to determine their adaptive value.

Interestingly, most (63%) population-differentiating accessory genes were present in some admixed isolates (Fig 6) and the vast majority (98%) were conserved to some degree in other *Fusarium* species (S2 Table), with a few exhibiting > 90% identity to homologs in distantly related species (e.g., *F*. *oxysporum*). These findings are in accordance with previous studies [23], indicating that accessory genes are often exchanged among *F*. *graminearum* and other species of fusaria, or they were present in the ancestral gene pool and were lost and/or duplicated in a population-specific manner. As such, standing variation in the *Fusarium* gene pool may provide a reservoir of interchangeable niche-specific traits that enhance the adaptive capacity of *F*. *graminearum* by facilitating rapid evolution [49].

### Functional implications of genomic divergence in FHB pathogens

Based on patterns of genomic divergence, it appears that *F*. *graminearum* cereal pathogens may rely on a small subset of rapidly evolving core genes, and an array of modular, accessory genes, to fine-tune the genome in response to selection. This genomic architecture has allowed for the evolution of divergent gene sets within populations (Fig 4), which in turn, could contribute to differences in the functional capacity of sympatric FHB pathogens. Given the strong selective constraints imposed by agricultural landscapes [6], it was not surprising that many of the population-specific candidate genes encoded pathogenicity-related proteins. Previous studies identified 55 candidate virulence genes that were unique to the PH-1 reference strain [23], however, 95% of these genes were prevalent in the genomes sampled herein (found in 38% of isolates on average), and only two showed patterns of conservation that were suggestive of population-specific adaptations. The genomic features identified in our study, on the other hand, provided evidence that populations could be differentiated by numerous gene variants and combinations of accessory genes encoding proteins involved in pathogenicity. These included plant cell-wall degrading enzymes, major facilitator transporters and secreted effector proteins (Table 2, Fig 6, S2 Table), which are known to be expressed *in planta*, and may be associated with different strategies for host invasion and nutrient acquisition from degraded plant tissues [22,36,50–52].

The FHB pathogens in our study were known to produce different analogs of trichothecene mycotoxins [11,17,19], which can affect virulence [13,53]. However, we uncovered additional population-level diversity in putative secondary metabolite-related genes, including four previously undescribed gene clusters, multiple genes encoding NRPS enzymes, and several cytochrome P450 enzymes (Fig 6, S2 Table). For instance, a uniquely conserved putative gene cluster in the NA2 population encoded a fungal transcription factor, a monoterpene hydrolase and a salicylate hydroxylase. In other studies, salicylate hydrolases were shown to degrade the plant defense hormone, salicylic acid [54,55]. On the other hand, we found evidence of broad diversification in oxidoreductive gene function in the NA3 population (Fig 4), which was uniquely associated with a previously undescribed secondary metabolite gene cluster showing high similarity (91–93% identity) to genes in *F*. *oxysporum* (S2 Table, Y). Although these genes have not been functionally characterized, based on similarity to secondary metabolite genes in fungal pathogens, we speculate that many may be involved in the biosynthesis of metabolites that function as plant effectors and virulence factors [56–61]. Thus, the three populations may employ different metabolites during infection [62], or utilize different virulence factors to avoid host recognition [63].

Our findings also provided evidence that competition has imposed strong selective constraints on NA1, NA2 and NA3 isolates, resulting in population-level divergence in genes that allow the fungus to detect and respond to antagonists. *F*. *graminearum* populations could be differentiated by multiple heterokaryon incompatibility genes (*het*), and genes encoding NACHT, STAND, NB-ARC and HELO domains (Table 2, Fig 6, S2 Table) involved in non-self recognition and heterokaryon cell death, [40,64,65]. In addition, we detected population-level divergence in antimicrobial effectors, including an anti-fungal toxin [32], a secreted virucidal protein [66] (Table 2) and a putative antibiotic biosynthetic gene cluster (S2 Table, F). For example, different protein variants of Hce2 chitinase, an effector that likely functions to inhibit fungal competitors [32], were found in the three populations, and in NA3 there was strong evidence that selection has resulted in the fixation of a distinct variant of Hce2 (Table 2). Population-level divergence of incompatibility genes could indicate that these loci contribute to reproductive isolation [67], though patterns of trans-species polymorphism at non-self recognition and fungal immunity genes in FGSC species (Table 2, FGRRES_10558, FGRRES_12215, FGRRES_13758) could indicate balancing or frequency-dependent selection to maintain diversity in nature [68–71]. Taken together, divergence of toxigenic peptides and non-self recognition proteins suggests that like other filamentous fungi, *F*. *graminearum* utilizes diverse extracellular antimicrobial toxins during antagonistic interactions [40]. In *F*. *verticillioides*, encounters with other fungi result in production of secondary metabolites that kill and inhibit growth of fungal competitors [72]. In the wild, *F*. *graminearum* co-occurs with other microbes, including other *Fusarium* species [73–75]. Several studies have shown that *F*. *graminearum* interacts competitively with other *Fusarium* species *in vitro* and in the field [73,76], and *in planta*, intraspecific interactions can inhibit mycotoxin production and pathogenicity [23]. Therefore, it is conceivable that *F*. *graminearum* populations differ in their ability to interact competitively, which could impact FHB pathogen composition and diversity of the cereal microbiome.

Although environmental factors, particularly temperature, have been shown to influence the distribution of FGSC species, there appear to be few climatic constraints on *F*. *graminearum* [77]. However, there is evidence that certain isolates have enhanced cold tolerance. For example, some isolates with the 3ADON chemotype appear to be more resistant to freezing than 15ADON isolates [74], which may confer an advantage to certain populations of the fungus in cooler climates [73]. Herein, we found that populations of *F*. *graminearum* have evolved unique genomic traits that could influence how these pathogens respond to environmental factors. First, in NA2 we found strong evidence of a selective sweep targeting a locus encoding Pgl1, a polyketide synthase involved in pigment production [37], a DEAD helicase (FGRRES_13527), and a putative plant effector (FGRRES_09181) [36] (Fig 3A). The discovery of a DEAD helicase was striking, because homologs of this gene have been implicated in the adaptive evolution of multiple fungal species [44,45], and are essential for local adaptation to climate in *N*. *crassa* [44]. Due to the close linkage of these genes, it is unclear which was targeted by selection. However, parallel findings in other fungal species strongly support the hypothesis that divergence of the DEAD helicase gene in populations of *F*. *graminearum* reflects adaptation to different local conditions. This may indicate that sympatric populations evolved in different environments or have different environmental optima.

NA1 also showed evidence of significant divergence at genomic regions involved in cold adaptation, as this population was uniquely associated with a homolog of the cold shock protein Crp1, located within a putative plant effector biosynthetic gene cluster (S2 Table; J). Crp1 is a multifaceted RNA chaperone that promotes cold tolerance, by stabilizing RNA intermediates at suboptimal temperatures, but also regulates fungal pathogenicity [78]. We posit that this variant may perform similar functions within the NA1 genomic background. We also identified an ortholog of the circadian clock gene *frequency* (*frq*) that was only conserved in NA1 (FGRRES_08069 Fig 6, S2 Table). Recent genome scans identified this gene as a driver of local adaptation in *N*. *crassa*, presumably because it allows the fungus to adjust circadian oscillations in response to photoperiod [44]. A *frq-*regulated circadian clock has not been described in *F*. *graminearum*, but previous studies reported a single ortholog within the genome [79] (FGRRES_06054; 3:4,152,665–4,155,702). We found orthologs of this gene that exhibited 99–100% identity in all *F*. *graminearum* genomes examined in this study, indicating that it is part of the core genome. In contrast, FGRRES_08069 was located on a different chromosome (2:305,728–306,903), and while it was highly conserved in NA1 isolates, it was found in only one NA2 isolate (Fig 6), likely reflecting the history of *frq* loss and duplication in *F*. *graminearum* [79]. Alignment with the *N*. *crassa* protein indicated low identity (34%), but revealed that the coil-coiled domain essential for FRQ function [80] was intact. Knowing that *N*. *crassa* utilizes different isoforms of FRQ to fine-tune clock-regulated protein expression in response to environmental stimuli [81], it is possible that the *frq* variant found in NA1 may also be involved in light or temperature sensitivity.

The NA3 population exhibited a selective sweep targeting a different circadian regulator, a DASH cryptochrome, which colocalized with genes encoding a kinase and a methylcitrate dehydratase (Table 2). DASH cryptochromes are blue/UV-A light photoreceptors and DNA repair enzymes that have been shown to play essential roles in regulating stress response and fungal growth [82]. In *F*. *fujikuroi*, the DASH cryptochrome, CryD, also regulates production of secondary metabolites, such as pigments, and the plant hormone, gibberellin, in a light-dependent manner [33]. The cryptochrome in NA3 was distantly related to the *F*. *fujikuroi* CryD homolog (64% identical) and exhibited considerable divergence from the reference *F*. *graminearum* CryD homolog (89% identical), but was highly similar to an ortholog found in *F*. *culmorum* (94% identity), an FHB pathogen found in the same geographic areas as the NA3 population [19]. Further analyses of the adaptive significance of the NA3 CryD ortholog are warranted, but our findings may indicate non-phylogenetic sorting of this gene, perhaps reflecting selection or recent genetic introgression at this locus.

Our comparative genomics study suggests that there may be marked ecological diversity among sympatric NA1, NA2 and NA3 *F*. *graminearum*, likely reflecting the unique demographic histories and population-specific selection pressures experienced by independently evolving populations of the fungus. All three populations had distinct genomic traits that could provide a selective advantage under specific ecological scenarios. Specifically, the outliers found on chromosome 2 that were associated with known quantitative trait polymorphisms in *F*. *graminearum* provide strong evidence that populations have divergent fitness traits involved in fungicide resistance and mycotoxin-associated virulence. Many of the population-differentiating genes appeared to be involved in host-infection, however, it remains to be determined if they evolved as adaptations to wild grasses, domesticated cereals or other plant hosts. Regardless, like other phytopathogens, *F*. *graminearum* exhibits evidence of genome evolution in response to the constant arms-race between pathogen and host [62,63]. Yet, standing variation in the *Fusarium* gene pool seems to be an important source of divergent alleles, and potentially, a reservoir of adaptations maintained in different populations through balancing selection. The undeniable parallels between candidate genomic adaptations identified herein and those found in saprophytic and ectomycorrhizal fungi, suggests commonalities in the genetic bases of adaptive population divergence, such as the importance of circadian regulators and helicases in adaptation to light and temperature [44,83]. In the future, the candidate genes identified in this study will enable functional testing of specific hypotheses about the genes that have contributed to the evolutionary dynamics of FHB pathogens, and also provide the foundation for further investigations of adaptive evolution in other fungal species.

## Conclusions

By combining high-resolution genome scans with surveys of gene content, we were able to identify genes involved in population divergence of a major fungal phytopathogen. Our results provided compelling evidence that population-specific selection pressures have left distinct signatures in the genomes of *F*. *graminearum* isolates, resulting in differentiation of an array of genes involved in host invasion, antagonism and environmental adaptation. The diffusion of adaptations among populations via localized gene flow or transcontinental introductions [11] could present future challenges to FHB management, because selection could continue to drive emergence of highly adapted, virulent pathogens. In this regard, persistent surveillance at a global scale will be critical for monitoring the influx of pathogens with novel adaptations and assessing the sustainability of resistant cultivars and fungicides used to control FHB. Moreover, gene deletion analyses of candidate traits *in planta* could provide insight into ecological significance of the pathogenicity-related genes identified herein, while direct competition experiments between different populations or species will allow us to reevaluate fungal effectors in regards to antagonistic interactions and microbial diversity. However, future studies need to consider that fitness differences in *F*. *graminearum* could also be related to gene expression or transcription regulation [84,85], and probably involve a multitude of quantitative traits that depend on host genotype, climate and the microbiome. As such, comparative genomics and transcriptomic studies of FHB populations sampled across space and time could be an efficient way to understand genome-wide adaptation in relation to specific climatic and agronomic factors.

## Materials and methods

### Genome sequencing

Sixty *F*. *graminearum* isolates collected from cereal crops in the northern US and southern Canada were selected for population genomic analyses (S1 Table). We included 20 isolates with the NA1 genetic background and the 15ADON chemotype (NA1/15ADON), 20 isolates with the NA2 genetic background and the 3ADON chemotype (NA2/3ADON) [19,20] and 20 isolates with the NX-2 chemotype [16]. Chemotypes were determined previously using multi-locus genotyping (MLGT) or gas chromatography mass spectrophotometry (GC-MS) [9,11,16,19,20], and all NA1 and NA2 *F*. *graminearum* were previously assigned to their respective populations with high confidence (probability of membership ≥ 99%) using RFLP or VNTR genotyping [9,11,19,20]. For reference, we also sampled isolates representing three closely related *Fusarium* species found in North America, *F*. *boothii* (*N =* 5), *F*. *gerlachii* (*N =* 3) and *F*. *louisianense* (*N =* 2), and an outgroup species of the FGSC, *F*. *pseudograminearum* (*N =* 1).

Fungal cultures were prepared for DNA extraction following methods described in O’Donnell et al. [86]. DNA from each isolate was quantified using the Quant-iT™ Qubit™ dsDNA HS Assay Kit (Invitrogen) and diluted to a final concentration of 1ng for library preparation with the Illumina Nextera XT DNA Library Prep Kit, following manufacturer’s instructions for 2x300bp MiSeq runs. All genomic data generated herein were deposited in the NCBI Sequence Read Archive (Accession numbers SRR5948910-SRR5948979, SRR6396633). Before analysis, sequencing reads were trimmed to remove adaptors, low quality (Q < 20) bases and ambiguous nucleotides, and reads were filtered to eliminate bacterial and human DNA contaminants.

### Reference-based mapping and *de novo* assembly

We used CLC Genomics Workbench version 9.5.2 (https://www.qiagenbioinformatics.com/) to map reads from all isolates to the genome sequence of the *F*. *graminearum* reference strain, PH-1 (Accession no. PRJEB5475), using gene and domain annotations from King et al. (2015) (S1 File). After mapping, duplicate reads were removed and local realignment was performed to resolve gaps and indels. To characterize genomic regions that were too divergent to be mapped (< 80% identity over > 50% of the read) or were not represented in the reference genome, we also performed *de novo* assembly of unmapped reads using default parameters in CLC Genomics Workbench, and then applied filters to remove regions with less than 5X coverage per genome. Augustus version 3.2 [87] was used to employ a Hidden Markov model to predict genes on these contigs, utilizing validated parameters settings based on experimentally validated introns from *F*. *graminearum* [88]. Coding sequences were identified on both strands of DNA, requiring ATG start codons and stop codons. Herein we refer to contigs assembled from unmapped reads as orphan contigs, and genes annotated on orphan contigs as orphan genes.

### SNP discovery

We used the Genome Analysis Tool Kit [89] and followed best practices for genomic variant discovery [90] to recalibrate base-quality scores of read mappings and perform multi-sample SNP discovery. Indels were not considered as population genomic analyses focused on methods that utilized models of nucleotide substitution. Previously published, known sites of variation identified in the genome of PH-1 [22] were used to guide base-quality score recalibration and SNP validation. SNP calls with a Phred quality score < 30 or a mapping quality score < 20 in single samples were not considered during multi-sample SNP identification. In addition, we performed variant-quality score recalibration, utilizing filters to remove variants that exhibited strand bias, low quality or low read depth per genome, and ensuring that SNP sites included in genomic analyses had an estimated false discovery rate (FDR) <1%. To avoid ascertainment bias associated with sampling depth, we limited our analyses to sites having valid SNP calls in all samples [91].

### Population genomic structure

Bayesian clustering analyses implemented in the program STRUCTURE [92] were used to establish genome-level demographic structure, discern the population identity of NX-2 *F*. *graminearum* and examine admixture among NA1, NA2 and NX-2 isolates. To minimize the effects of linkage disequilibrium and reduce computational load, we limited analyses to SNPs that were at least 5 kb apart (6,852 sites) as pilot analyses indicated that this sampling interval provided sufficient resolution to confidently assess population structure and admixture. We simulated *K*, the number of populations, accounting for shared ancestry by utilizing the linkage model [93], and used 50,000 Monte Carlo Markov Chain (MCMC) iterations following a 20,000 iteration burn-in for each of three replicate runs performed for *K* 1–7. The model that maximized Δ*K*, the rate of change in log likelihood values, was considered optimal based on methods described in Evanno et al. [94]. Estimates of *q*, the proportion of each isolate’s membership in each of the inferred populations, were calculated using the optimal model parameters to assess probability of assignment and genetic admixture among populations.

Phylogenetic trees were generated from the full set of quality-filtered SNPs using rapid bootstrap [95] and maximum likelihood methods implemented in RAxML version 8.0.26 [96]. The General-Time-Reversible (GTR) model of nucleotide substitution, with corrections for ascertainment bias [97] and rate heterogeneity (ASC_GTRGAMMA), was used to infer a phylogeny of FGSC species. The SNPs identified after reference-based mapping (methods described above) of genome sequences from isolates of *F*. *boothii*, *F*. *gerlachii* and *F*. *louisianense* were included as outgroups in this tree. In addition, we built a phylogenetic network of *F*. *graminearum* isolates using SplitsTree4 version 4.14.6 [98] to depict an evolutionary history that reflected intraspecific recombination events.

### High-resolution genome scans for selection

To identify regions that have been impacted by selection in the NA1, NA2 and NA3 populations we calculated several population genetic summary statistics in non-overlapping, 10 kb sliding windows using the R package PopGenome [99]. We chose 10 kb intervals based on patterns of linkage disequilibrium in the *F*. *graminearum* genome, which become negligible at distances > 10 kb [42]. Further, pilot analyses exploring a range of window sizes (5 kb–20 kb) indicated that a 10 kb resolution generally captured covarying signals from linked loci, while distinguishing signals from adjacent loci in linkage equilibrium.

We quantified within-population diversity by calculating *π* (nucleotide diversity, the average number of differences between individuals, [100]), *θ* (nucleotide polymorphism based on segregating sites, [101]) and Tajima’s *D* [30], and estimated the number of recombination events within a population using *R*_*M*_ (four-gamete test, [102]). In addition we quantified differentiation between populations by calculating pairwise values of *D*_*xy*_ (nucleotide divergence based on the absolute number of differences between two populations, [100,103]), *F*_*ST*_ ([104], per haplotype, adjusted for unequal population sizes using weighted averages), and Tajima’s *D*, in this case calculated for combined populations as a relative measure of inter-population divergence [44]. Genome-wide averages of summary statistics were compared among the three populations using JMP [105], and visualized using Circos [106] and JalView Version 2 [107].

Genomic patterns created by genetic structuring, bottlenecks and population expansions can mimic those elicited by selection [25,108–110], thus it was important to assess the significance of outliers against the genomic background of our populations to discriminate selective events targeting individual loci from neutral demographic processes that impact the entire genome [111]. However, the mixed reproductive system and introgression documented in *F*. *graminearum* [42,112], coupled with notable trans-species polymorphism [15,16] and horizontal transfer [113–115] in *Fusarium* species violate assumptions and introduce severe bias in coalescent and likelihood-based demographic models used for detecting selection [116,117]. As such, we adapted a nonparametric permutation method for genome-wide SNP data [118] to evaluate the significance of 10 kb sliding-window statistics against the genomic distribution of values for each population. This method has proven effective for identifying population-specific outlier loci in species with complex evolutionary histories, as it utilizes localized patterns of linkage disequilibrium to discern extreme patterns of genetic variation elicited by natural selection from those elicited by neutral demographic processes [111,118]. We used custom shell scripts and BedTools [119] to generate 1000 random permutations of the variant call format (VCF) file, containing the chromosomal coordinates of each SNP and the associated genotypes observed for the 60 sequenced isolates. Each permutation randomized observed genotypes across the SNP coordinates, generating a random set of SNP coordinate/genotype combinations that maintained the joint site-frequency spectrum of the data, but was unconstrained by linkage disequilibrium (i.e. spatially randomized). Sliding-window analyses were repeated on each of the 1000 permuted datasets as described above to generate a null genome-wide distribution of summary statistics for each population, and all pairwise combinations of populations. Observed values for a 10 kb window were considered outliers at *P <* 0.001, i.e. the probability that the observed value was more extreme than values from the null distribution. As such, outliers were identified as those windows that exhibited localized signatures of linkage disequilibrium associated with selective sweeps, and had patterns of variation that were significant in the context of each population’s genome-wide diversity. Still, to reduce the probability of identifying false positives, genomic regions of interest were limited to those having significant values of absolute and relative differentiation between population pairs [44] (significant *F*_*ST*_, *D*_*xy*_, and Tajima’s *D* in at least two population comparisons), coupled with significant reductions in nucleotide diversity (*π*, within the targeted population).

Phylogenetic analyses were performed on genes residing in outlier regions using sequences from *F*. *pseudograminearum* as an FGSC outgroup for *F*. *graminearum*, *F*. *boothii*, *F*. *gerlachii* and *F*. *louisianense*. DNA sequences were aligned with MUSCLE (Edgar, 2004) as implemented in CLC Genomics Workbench, and then maximum likelihood phylogenies were constructed using IQtree [120], with models of molecular evolution selected on the basis of Bayesian Information Criterion scores calculated with ModelFinder [121]. Support for individual branches was assessed using 1000 ultrafast bootstraps [122]. We also examined scenarios of trans-species evolution by comparing the likelihood of unconstrained trees to constraint trees requiring monophyly of all *F*. *graminearum* isolates using Shimodaira-Hasegawa likelihood ratio tests [123].

### Quantifying differences in gene content

To compare gene content among isolates we conducted an inventory of all *F*. *graminearum* genes, including coding sequences extracted from read mappings and genes predicted on orphan contigs. Annotated PH-1 gene sequences were also included as an internal reference. We first identified orthologous gene sequences using R-functions within the micropan package [124]. All-against-all BLAST comparisons were conducted on predicted protein sequences and then pairwise distance values (*D*_*i*,*j*_) were computed from BLAST alignment scores (bitscores). Sequences were allocated into orthologous groups using single linkage clustering with *D*_*i*,*j*_ ≥ 0.5 set as the cluster inclusion threshold, as this allowed us to resolve 97% of the PH-1 genes as distinct homologs (i.e. a one to one match between annotated genes and ortholog groups). A pan-genome matrix indicating the number of copies of each ortholog per genome was then constructed based on sequence clustering. The presence/absence of orthologous genes in each genome was verified by examining BLAST alignments and gene coverage in individual read mappings. Genes encoding proteins that were present in the reference genome and in all sampled genomes were classified as core genes, whereas genes encoding proteins that were missing in one or more genomes were classified as accessory genes. To confirm that core genes were not misclassified as accessory genes using BLAST-based similarity searches, we also classified orthologs based on Pfam domain content and order, using HMMER version 3.1 (http://hmmer.org) to characterize domain profiles for all accessory proteins.

To identify genes that were differentially conserved across populations, we used a gene enrichment test adapted from den Bakker et al. [125] to compare the relative frequency of each accessory gene in the three populations. Genes were considered differentially conserved when the test statistic (*E*) exceeded three standard deviations of the population mean, i.e., an empirical *P-*value < 0.01.

A dendrogram of the *F*. *graminearum* pan-genome was computed using the neighbor joining method implemented in MEGA 7 [126] and pairwise distances calculated between genomes based on gene presence/absence polymorphism.

### Functional annotation and enrichment analyses

BLAST2GO version 3.2.7 [127,128] was used to assign enzyme codes, InterPro domains and Gene Ontology (GO) terms to proteins predicted from differentially conserved genes. Putative functions and homologues of these proteins were further explored using BLAST [129] and CDD [130,131] database searches. We also identified secreted proteins that lacked a transmembrane domain using SignalP version 4.1 [132]. Blast searches of PHI-base (www.PHI-base.org) were also conducted to examine homology with functionally characterized virulence genes in *F*. *graminearum* and other phytopathogenic microbes.

Gene Ontology (GO) enrichment analyses were performed on the candidate genes identified in each population (differentially conserved genes and genes located within outliers) using the R package XGR version 1.0.4 [133]. One-sided hypergeometric tests were performed to compare the relative frequency of GO terms assigned to domains in candidate gene sets to GO terms assigned to domains from all *F*. *graminearum* genes, requiring that GO terms have >3 hits in common between candidate and reference lists to account for the smaller number of genes analyzed in population-specific gene sets. In addition, we utilized a stringent criteria for significance by applying the Benjamini-Hochberg method to adjust for multiple tests, and identifying GO terms as significantly overrepresented only when the adjusted *P*-value for that term was < 0.01. Simulation studies indicated that these analytical conditions result in a low false positive rate (< 0.01) for datasets with sample sizes comparable to those analyzed herein [133].

## Supporting information

S1 Fig Maximum likelihood phylogeny of *TRI1* gene sequences from the 60 isolates of *F*. *graminearum* used for genome sequencing. The phylogeny was inferred using the Kimura 2-parameter model of nucleotide substitution [135] with a Gamma parameter to account for rate heterogeneity. Bootstrap values (%, based on 100 replications) ≥ 50 are indicated on branches. The tree was rooted at midpoint and drawn to scale, with branch lengths measured in the number of substitutions per site.
(DOCX)

S2 Fig Outlier gene phylogenies for *F*. *graminearum*, *F*. *gerlachii*, *F*. *louisianense*, *F*. *boothii* and *F*. *pseudograminearum*. Maximum likelihood methods were used to construct phylogenies for 75 of the 81 genes residing in genomic regions exhibiting signatures of selection. The remaining six genes were not analyzed because homologs were not detected in one or more of the *F*. *graminearum* outgroup species (*F*. *gerlachii*, *F*. *louisianense*, *F*. *boothii* or *F*. *pseudograminearum*). Above, outlier genes located on chromosome 1, between 11.30 to 11.31 Mb are shown to exemplify discordance between phylogenies of outlier genes and the genome-wide SNP phylogeny (Fig 1). Numbers on branches indicate support values determined with 1000 ultrafast bootstraps [120,122]. The tree was rooted with *F*. *pseudograminearum*, a member of the *Fusarium sambucinum* species complex and a basal outgroup to the FGSC [26]. Branch lengths are drawn to scale and indicate the number of substitutions per site.
(DOCX)

S3 Fig Genome-wide phylogeny of *F*. *graminearum* and related FGSC species inferred in the absence of outlier regions. Maximum likelihood methods were used to construct a phylogeny from SNPs as in Fig 1B, except sites found in outlier regions showing significant evidence of selection were omitted. All bootstrap values were 100%, except those indicated at branch nodes. The tree was rooted with *F*. *boothii* and drawn to scale, with branch lengths measured in the number of substitutions per site.
(DOCX)

S4 Fig Frequency distribution of *F*. *graminearum* accessory genes. Reference-mapped genes and orphan genes were clustered into orthologous groups based on BLAST all-versus-all comparisons of predicted protein sequences. Gene frequency was quantified based on the presence/absence of each ortholog in the 60 sampled genomes and the PH-1 reference genome. The frequency of accessory genes (orthologs found in a subset of isolates, *N =* 1,681) is shown above. Core genes (*N =* 13,632) found in all 61 genomes are not depicted in the figure.
(DOCX)

S1 File Ensembl mapping file of gene annotations for *Fusarium graminearum* str. PH-1. (TXT)

S2 File Predicted sequences for orphan proteins based on inferred gene models. (FASTA)

S1 Table *Fusarium* isolates chosen for genome sequencing. (XLSX)

S2 Table Differentially conserved accessory genes. ^1^Protein functions were inferred using BLAST, CDD [130,131], and InterPro database searches and analyses conducted with BLAST2GO version 3.2.7 [127,128]. Asterisks indicate secreted proteins identified using SignalP 4.1 [132]. PHI-base accession numbers are indicated in parentheses for sequences showing significant homology (BLAST *e-*value < 10^−5^) to proteins in the PHI-base 4.1 database. ^2^ Ortholog had a significant (*e-*value < 10^−5^) BLAST hit to another species, or to another ortholog in PH-1. Only the top hits (based on bitscore) are shown. ^3^ Genes with the same designation were clustered on the same contig, or colocalized within the PH-1 genome.
(XLSX)

## References

[1] Goswami RS, Kistler HC (2004) Heading for disaster: *Fusarium graminearum* on cereal crops. Molecular Plant Pathology 5: 515–525. doi: 10.1111/j.1364-3703.2004.00252.x 20565626 10.1111/j.1364-3703.2004.00252.x

[2] McMullen M, Bergstrom G, De Wolf E, Dill-Macky R, Hershman D, et al (2012) A unified effort to fight an enemy of wheat and barley: Fusarium head blight. Plant Disease 96: 1712–1728. 10.1094/PDIS-03-12-0291-FE 30727259

[3] Desjardins A, Proctor R (2007) Molecular biology of *Fusarium* mycotoxins. International Journal of Food Microbiology 119: 47–50. doi: 10.1016/j.ijfoodmicro.2007.07.024 17707105 10.1016/j.ijfoodmicro.2007.07.024

[4] Dubin H, Gilchrist L, Reeves L, McNab A (2002) Fusarium head blight: global status and prospects In: Curtis BR, S; Macpherson HG;, editor. Bread wheat: improvement and production. Rome, Italy: Food and Agriculture Organization of the United Nations.

[5] Nganje WE, Kaitibie S, Wilson WW, Leistritz FL, Bangsund DA (2004) Economic impacts of Fusarium head blight in wheat and barley: 1993–2001. Agribusiness and Applied Economics Report No 538. ND, US: Department of Agribusiness and Applied Economics, North Dakota State University.

[6] McDonald BA, Stukenbrock EH (2016) Rapid emergence of pathogens in agro-ecosystems: global threats to agricultural sustainability and food security. Philosophical Transactions Royal Society B 371: 20160026. 10.1098/rstb.2016.0026 PMC5095548 28080995

[7] McMullen M, Jones R, Gallenberg D (1997) Scab of wheat and barley: a re-emerging disease of devastating impact. Plant Disease 81: 1340–1348. 10.1094/PDIS.1997.81.12.1340 30861784

[8] Zeller KA, Bowden RL, Leslie JF (2003) Diversity of epidemic populations of Gibberella zeae from small quadrats in Kansas and North Dakota. Phytopathology 93: 874–880. doi: 10.1094/PHYTO.2003.93.7.874 18943169 10.1094/PHYTO.2003.93.7.874

[9] Gale L, Ward T, Balmas V, Kistler H (2007) Population subdivision of *Fusarium graminearum* sensu stricto in the upper Midwestern United States. Phytopathology 97: 1434–1439. doi: 10.1094/PHYTO-97-11-1434 18943513 10.1094/PHYTO-97-11-1434

[10] Schmale D, Wood‐Jones A, Cowger C, Bergstrom G, Arellano C (2011) Trichothecene genotypes of *Gibberella zeae* from winter wheat fields in the eastern USA. Plant Pathology 60: 909–917.

[11] Ward TJ, Clear RM, Rooney AP, O’Donnell K, Gaba D, et al (2008) An adaptive evolutionary shift in Fusarium head blight pathogen populations is driving the rapid spread of more toxigenic *Fusarium graminearum* in North America. Fungal Genetics and Biology 45: 473–484. doi: 10.1016/j.fgb.2007.10.003 18035565 10.1016/j.fgb.2007.10.003

[12] Puri KD, Zhong S (2010) The 3ADON population of *Fusarium graminearum* found in North Dakota is more aggressive and produces a higher level of DON than the prevalent 15ADON population in spring wheat. Phytopathology 100: 1007–1014. doi: 10.1094/PHYTO-12-09-0332 20839936 10.1094/PHYTO-12-09-0332

[13] Jansen C, Von Wettstein D, Schäfer W, Kogel K-H, Felk A, et al (2005) Infection patterns in barley and wheat spikes inoculated with wild-type and trichodiene synthase gene disrupted *Fusarium graminearum*. Proceedings of the National Academy of Sciences of the United States of America 102: 16892–16897. doi: 10.1073/pnas.0508467102 16263921 10.1073/pnas.0508467102 PMC1283850

[14] Foroud N, McCormick S, MacMillan T, Badea A, Kendra D, et al (2012) Greenhouse studies reveal increased aggressiveness of emergent Canadian *Fusarium graminearum* chemotypes in wheat. Plant Disease 96: 1271–1279. 10.1094/PDIS-10-11-0863-RE 30727146

[15] Ward TJ, Bielawski JP, Kistler HC, Sullivan E, O'Donnell K (2002) Ancestral polymorphism and adaptive evolution in the trichothecene mycotoxin gene cluster of phytopathogenic *Fusarium*. Proceedings of the National Academy of Sciences 99: 9278–9283. 10.1073/pnas.142307199 PMC123131 12080147

[16] Kelly AC, Proctor RH, Belzile F, Chulze S, Clear RM, et al (2016) The geographic distribution and complex evolutionary history of the NX-2 trichothecene chemotype from *Fusarium graminearum*. Fungal Genetics and Biology 95: 39–48. doi: 10.1016/j.fgb.2016.08.003 27497828 10.1016/j.fgb.2016.08.003

[17] Liang J, Lofgren L, Ma Z, Ward TJ, Kistler HC (2015) Population subdivision of *Fusarium graminearum* from barley and wheat in the Upper Midwestern United States at the turn of the century. Phytopathology 105: 1466–1474. doi: 10.1094/PHYTO-01-15-0021-R 26107972 10.1094/PHYTO-01-15-0021-R

[18] Varga E, Wiesenberger G, Hametner C, Ward TJ, Dong Y, et al (2015) New tricks of an old enemy: Isolates of *Fusarium graminearum* produce a type A trichothecene mycotoxin. Environmental Microbiology 17: 2588–2600. doi: 10.1111/1462-2920.12718 25403493 10.1111/1462-2920.12718 PMC4950012

[19] Kelly AC, Clear RM, O’Donnell K, McCormick S, Turkington K, et al (2015) Diversity of Fusarium head blight populations and trichothecene toxin types reveals regional differences in pathogen composition and temporal dynamics. Fungal Genetics and Biology 82: 22–31. doi: 10.1016/j.fgb.2015.05.016 26127017 10.1016/j.fgb.2015.05.016

[20] Liang J, Xayamongkhon H, Broz K, Dong Y, McCormick SP, et al (2014) Temporal dynamics and population genetic structure of *Fusarium graminearum* in the upper Midwestern United States. Fungal Genetics and Biology 73: 83–92. doi: 10.1016/j.fgb.2014.10.002 25312860 10.1016/j.fgb.2014.10.002

[21] Lofgren L, Riddle J, Dong Y, Kuhnem PR, Cummings JA, et al (2017) A high proportion of NX-2 genotype strains are found among *Fusarium graminearum* isolates from northeastern New York State. European Journal of Plant Pathology: 1–6.

[22] Cuomo CA, Güldener U, Xu J-R, Trail F, Turgeon BG, et al (2007) The *Fusarium graminearum* genome reveals a link between localized polymorphism and pathogen specialization. Science 317: 1400–1402. doi: 10.1126/science.1143708 17823352 10.1126/science.1143708

[23] Walkowiak S, Bonner CT, Wang L, Blackwell B, Rowland O, et al (2015) Intraspecies interaction of *Fusarium graminearum* contributes to reduced toxin production and virulence. Molecular Plant-Microbe Interactions 28: 1256–1267. doi: 10.1094/MPMI-06-15-0120-R 26125491 10.1094/MPMI-06-15-0120-R

[24] Smith JM, Haigh J (1974) The hitch-hiking effect of a favourable gene. Genetical Research 23: 23–35. 4407212

[25] Akey JM (2009) Constructing genomic maps of positive selection in humans: where do we go from here? Genome Research 19: 711–722. doi: 10.1101/gr.086652.108 19411596 10.1101/gr.086652.108 PMC3647533

[26] O'Donnell K, Rooney AP, Proctor RH, Brown DW, McCormick SP, et al (2013) Phylogenetic analyses of RPB1 and RPB2 support a middle Cretaceous origin for a clade comprising all agriculturally and medically important fusaria. Fungal Genetics and Biology 52: 20–31. doi: 10.1016/j.fgb.2012.12.004 23357352 10.1016/j.fgb.2012.12.004

[27] King R, Urban M, Hammond-Kosack MC, Hassani-Pak K, Hammond-Kosack KE (2015) The completed genome sequence of the pathogenic ascomycete fungus *Fusarium graminearum*. BMC Genomics 16: 1 doi: 10.1186/1471-2164-16-1 26198851 10.1186/s12864-015-1756-1 PMC4511438

[28] Laurent B, Moinard M, Spataro C, Ponts N, Barreau C, et al (2017) Landscape of genomic diversity and host adaptation in *Fusarium graminearum*. BMC Genomics 18: 203 doi: 10.1186/s12864-017-3524-x 28231761 10.1186/s12864-017-3524-x PMC5324198

[29] Stajich JE, Hahn MW (2005) Disentangling the effects of demography and selection in human history. Molecular Biology and Evolution 22: 63–73. doi: 10.1093/molbev/msh252 15356276 10.1093/molbev/msh252

[30] Tajima F (1989) Statistical method for testing the neutral mutation hypothesis by DNA polymorphism. Genetics 123: 585–595. 2513255 10.1093/genetics/123.3.585 PMC1203831

[31] Lu S, Edwards MC (2016) Genome-wide qnalysis of small secreted cysteine-rich proteins edentifies candidate effector proteins potentially involved in *Fusarium graminearum*−wheat interactions. Phytopathology 106: 166–176. doi: 10.1094/PHYTO-09-15-0215-R 26524547 10.1094/PHYTO-09-15-0215-R

[32] Stergiopoulos I, Kourmpetis YA, Slot JC, Bakker FT, De Wit PJ, et al (2012) In silico characterization and molecular evolutionary analysis of a novel superfamily of fungal effector proteins. Molecular Biology and Evolution 29: 3371–3384. doi: 10.1093/molbev/mss143 22628532 10.1093/molbev/mss143

[33] Castrillo M, Garcia-Martinez J, Avalos J (2013) Light-dependent functions of the *Fusarium fujikuroi* CryD DASH cryptochrome in development and secondary metabolism. Applied Environmental Microbiology 79: 2777–2788. doi: 10.1128/AEM.03110-12 23417004 10.1128/AEM.03110-12 PMC3623198

[34] Alexander NJ, Proctor RH, McCormick SP (2009) Genes, gene clusters, and biosynthesis of trichothecenes and fumonisins in *Fusarium*. Toxin Reviews 28: 198–215.

[35] Kimura M, Tokai T, O’Donnell K, Ward TJ, Fujimura M, et al (2003) The trichothecene biosynthesis gene cluster of *Fusarium graminearum* F15 contains a limited number of essential pathway genes and expressed non-essential genes. FEBS letters 539: 105–110. 12650935 10.1016/s0014-5793(03)00208-4

[36] Brown NA, Antoniw J, Hammond-Kosack KE (2012) The predicted secretome of the plant pathogenic fungus *Fusarium graminearum*: a refined comparative analysis. PLoS One 7: e33731 doi: 10.1371/journal.pone.0033731 22493673 10.1371/journal.pone.0033731 PMC3320895

[37] Gaffoor I, Brown DW, Plattner R, Proctor RH, Qi W, et al (2005) Functional analysis of the polyketide synthase genes in the filamentous fungus *Gibberella zeae* (anamorph *Fusarium graminearum*). Eukaryotic Cell 4: 1926–1933. doi: 10.1128/EC.4.11.1926-1933.2005 16278459 10.1128/EC.4.11.1926-1933.2005 PMC1287854

[38] Laurent B, Palaiokostas C, Spataro C, Moinard M, Zehraoui E, et al (2017) High‐resolution mapping of the recombination landscape of the phytopathogen *Fusarium graminearum* suggests two‐speed genome evolution. Molecular Plant Pathology. 10.1111/mpp.12524 PMC6638080 27998012

[39] Henriksson G, Johansson G, Pettersson G (2000) A critical review of cellobiose dehydrogenases. Journal of Biotechnology 78: 93–113. 10725534 10.1016/s0168-1656(00)00206-6

[40] Bidard F, Clavé C, Saupe SJ (2013) The transcriptional response to nonself in the fungus *Podospora anserina*. G3: Genes| Genomes| Genetics 3: 1015–1030. doi: 10.1534/g3.113.006262 23589521 10.1534/g3.113.006262 PMC3689799

[41] Lofgren LA, LeBlanc NR, Certano AK, Nachtigall J, LaBine KM, et al (2017) *Fusarium graminearum*: pathogen or endophyte of North American grasses? New Phytologist: 1–6. 10.1111/nph.14894 PMC5813145 29160900

[42] Talas F, McDonald BA (2015) Genome-wide analysis of *Fusarium graminearum* field populations reveals hotspots of recombination. BMC Genomics 16: 1 doi: 10.1186/1471-2164-16-1 26602546 10.1186/s12864-015-2166-0 PMC4659151

[43] Talas F, Kalih R, Miedaner T, McDonald BA (2016) Genome-wide association study identifies novel candidate genes for aggressiveness, deoxynivalenol production, and azole sensitivity in natural field populations of Fusarium graminearum. Molecular Plant-Microbe Interactions 29: 417–430. doi: 10.1094/MPMI-09-15-0218-R 26959837 10.1094/MPMI-09-15-0218-R

[44] Ellison CE, Hall C, Kowbel D, Welch J, Brem RB, et al (2011) Population genomics and local adaptation in wild isolates of a model microbial eukaryote. Proceedings of the National Academy of Sciences 108: 2831–2836. 10.1073/pnas.1014971108 PMC3041088 21282627

[45] Branco S, Bi K, Liao HL, Gladieux P, Badouin H, et al (2016) Continental‐level population differentiation and environmental adaptation in the mushroom *Suillus brevipes*. Molecular Ecology 26: 2063–2076. doi: 10.1111/mec.13892 27761941 10.1111/mec.13892 PMC5392165

[46] Badouin H, Gladieux P, Gouzy J, Siguenza S, Aguileta G, et al (2017) Widespread selective sweeps throughout the genome of model plant pathogenic fungi and identification of effector candidates. Molecular Ecology 26: 2041–2062. doi: 10.1111/mec.13976 28012227 10.1111/mec.13976

[47] Coleman ML, Chisholm SW (2010) Ecosystem-specific selection pressures revealed through comparative population genomics. Proceedings of the National Academy of Sciences 107: 18634–18639. 10.1073/pnas.1009480107 PMC2972931 20937887

[48] Cordero OX, Polz MF (2014) Explaining microbial genomic diversity in light of evolutionary ecology. Nature Reviews Microbiology 12: 263–273. doi: 10.1038/nrmicro3218 24590245 10.1038/nrmicro3218

[49] Barrett RD, Schluter D (2008) Adaptation from standing genetic variation. Trends in Ecology & Evolution 23: 38–44. 18006185 10.1016/j.tree.2007.09.008

[50] Paper JM, Scott‐Craig JS, Adhikari ND, Cuomo CA, Walton JD (2007) Comparative proteomics of extracellular proteins *in vitro* and *in planta* from the pathogenic fungus *Fusarium graminearum*. Proteomics 7: 3171–3183. doi: 10.1002/pmic.200700184 17676664 10.1002/pmic.200700184

[51] Wong P, Walter M, Lee W, Mannhaupt G, Münsterkötter M, et al (2011) FGDB: revisiting the genome annotation of the plant pathogen *Fusarium graminearum*. Nucleic Acids Research 39: D637–D639. doi: 10.1093/nar/gkq1016 21051345 10.1093/nar/gkq1016 PMC3013644

[52] Thatcher LF, Williams AH, Garg G, Buck S-AG, Singh KB (2016) Transcriptome analysis of the fungal pathogen *Fusarium oxysporum* f. sp. *medicaginis* during colonisation of resistant and susceptible *Medicago truncatula* hosts identifies differential pathogenicity profiles and novel candidate effectors. BMC Genomics 17: 860 doi: 10.1186/s12864-016-3192-2 27809762 10.1186/s12864-016-3192-2 PMC5094085

[53] Harris L, Desjardins AE, Plattner R, Nicholson P, Butler G, et al (1999) Possible role of trichothecene mycotoxins in virulence of *Fusarium graminearum* on maize. Plant Disease 83: 954–960. 10.1094/PDIS.1999.83.10.954 30841080

[54] Vlot AC, Dempsey DMA, Klessig DF (2009) Salicylic acid, a multifaceted hormone to combat disease. Annual Review of Phytopathology 47: 177–206. doi: 10.1146/annurev.phyto.050908.135202 19400653 10.1146/annurev.phyto.050908.135202

[55] Ambrose KV, Tian Z, Wang Y, Smith J, Zylstra G, et al (2015) Functional characterization of salicylate hydroxylase from the fungal endophyte *Epichloë festucae*. Scientific Reports 5: 10939 doi: 10.1038/srep10939 26055188 10.1038/srep10939 PMC4460724

[56] Hu G, Chen SH, Qiu J, Bennett JE, Myers TG, et al (2014) Microevolution during serial mouse passage demonstrates FRE3 as a virulence adaptation gene in *Cryptococcus neoformans*. MBio 5: e00941–00914. doi: 10.1128/mBio.00941-14 24692633 10.1128/mBio.00941-14 PMC3977352

[57] Siewers V, Viaud M, Jimenez-Teja D, Collado IG, Gronover CS, et al (2005) Functional analysis of the cytochrome P450 monooxygenase gene bcbot1 of *Botrytis cinerea* indicates that botrydial is a strain-specific virulence factor. Molecular Plant Microbe Interactions 18: 602–612. doi: 10.1094/MPMI-18-0602 15986930 10.1094/MPMI-18-0602

[58] Son H, Seo YS, Min K, Park AR, Lee J, et al (2011) A phenome-based functional analysis of transcription factors in the cereal head blight fungus, Fusarium graminearum. PLoS Pathogens 7: e1002310 doi: 10.1371/journal.ppat.1002310 22028654 10.1371/journal.ppat.1002310 PMC3197617

[59] Song XS, Xing S, Li HP, Zhang JB, Qu B, et al (2016) An antibody that confers plant disease resistance targets a membrane-bound glyoxal oxidase in *Fusarium*. New Phytologist 210: 997–1010. doi: 10.1111/nph.13806 26720747 10.1111/nph.13806

[60] Sweigard JA, Carroll AM, Farrall L, Chumley FG, Valent B (1998) *Magnaporthe grisea* pathogenicity genes obtained through insertional mutagenesis. Molecular Plant Microbe Interactions 11: 404–412. doi: 10.1094/MPMI.1998.11.5.404 9574508 10.1094/MPMI.1998.11.5.404

[61] Wight WD, Kim KH, Lawrence CB, Walton JD (2009) Biosynthesis and role in virulence of the histone deacetylase inhibitor depudecin from *Alternaria brassicicola*. Molecular Plant Microbe Interactions 22: 1258–1267. doi: 10.1094/MPMI-22-10-1258 19737099 10.1094/MPMI-22-10-1258

[62] Rutter WB, Salcedo A, Akhunova A, He F, Wang S, et al (2017) Divergent and convergent modes of interaction between wheat and *Puccinia graminis* f. sp. *tritici* isolates revealed by the comparative gene co-expression network and genome analyses. BMC Genomics 18: 291 doi: 10.1186/s12864-017-3678-6 28403814 10.1186/s12864-017-3678-6 PMC5389088

[63] Baltrus DA, Nishimura MT, Romanchuk A, Chang JH, Mukhtar MS, et al (2011) Dynamic evolution of pathogenicity revealed by sequencing and comparative genomics of 19 *Pseudomonas syringae* isolates. PLoS Pathogens 7: e1002132 doi: 10.1371/journal.ppat.1002132 21799664 10.1371/journal.ppat.1002132 PMC3136466

[64] Aanen DK, Debets AJ, Glass NL, Saupe SJ (2010) Biology and genetics of vegetative incompatibility in fungi Cellular and Molecular Biology of Filamentous Fungi: American Society of Microbiology pp. 274–288.

[65] Leslie JF (1993) Fungal vegetative compatibility. Annual Review of Phytopathology 31: 127–150. doi: 10.1146/annurev.py.31.090193.001015 18643765 10.1146/annurev.py.31.090193.001015

[66] Matei E, Louis JM, Jee J, Gronenborn AM (2011) NMR solution structure of a cyanovirin homolog from wheat head blight fungus. Proteins: Structure, Function, and Bioinformatics 79: 1538–1549. 10.1002/prot.22981 PMC3076533 21365681

[67] Nosil P, Funk DJ, Ortiz-Barrientos D (2009) Divergent selection and heterogeneous genomic divergence. Molecular Ecology 18: 375–402. doi: 10.1111/j.1365-294X.2008.03946.x 19143936 10.1111/j.1365-294X.2008.03946.x

[68] Debets AJ, Dalstra HJ, Slakhorst M, Koopmanschap B, Hoekstra RF, et al (2012) High natural prevalence of a fungal prion. Proceedings of the National Academy of Sciences 109: 10432–10437. 10.1073/pnas.1205333109 PMC3387057 22691498

[69] Wu J, Saupe SJ, Glass NL (1998) Evidence for balancing selection operating at the het-c heterokaryon incompatibility locus in a group of filamentous fungi. Proceedings of the National Academy of Sciences 95: 12398–12403. 10.1073/pnas.95.21.12398 PMC22843 9770498

[70] Muirhead CA, Glass NL, Slatkin M (2002) Multilocus self-recognition systems in fungi as a cause of trans-species polymorphism. Genetics 161: 633–641. 12072460 10.1093/genetics/161.2.633 PMC1462126

[71] Zhao J, Gladieux P, Hutchison E, Bueche J, Hall C, et al (2015) Identification of allorecognition loci in *Neurospora crassa* by genomics and evolutionary approaches. Molecular Biology and Evolution 32: 2417–2432. doi: 10.1093/molbev/msv125 26025978 10.1093/molbev/msv125 PMC4540973

[72] Estrada AER, Hegeman A, Kistler HC, May G (2011) In vitro interactions between *Fusarium verticillioides* and *Ustilago maydis* through real-time PCR and metabolic profiling. Fungal Genetics and Biology 48: 874–885. doi: 10.1016/j.fgb.2011.06.006 21703356 10.1016/j.fgb.2011.06.006

[73] Xu X-M, Parry D, Nicholson P, Thomsett M, Simpson D, et al (2005) Predominance and association of pathogenic fungi causing Fusarium ear blight in wheat in four European countries. European Journal of Plant Pathology 112: 143–154.

[74] Duveiller E, Singh P, Mezzalama M, Singh R, Dababat A (2012) Wheat diseases and pests: a guide for field identification In: CIMMYT, editor. Mexico, DF: CIMMYT.

[75] Li P, Lin Y, Zhang H, Wang S, Qiu D, et al (2016) Molecular characterization of a novel mycovirus of the family Tymoviridae isolated from the plant pathogenic fungus *Fusarium graminearum*. Virology 489: 86–94. doi: 10.1016/j.virol.2015.12.004 26744993 10.1016/j.virol.2015.12.004

[76] Wagacha JM, Oerke E-C, Dehne H-W, Steiner U (2012) Interactions of *Fusarium* species during prepenetration development. Fungal Biology 116: 836–847. doi: 10.1016/j.funbio.2012.05.001 22749170 10.1016/j.funbio.2012.05.001

[77] Backhouse D (2014) Global distribution of *Fusarium graminearum*, *F*. *asiaticum* and F. *boothii* from wheat in relation to climate. European Journal of Plant Pathology 139: 161–173.

[78] Fang W, St Leger RJ (2010) RNA binding proteins mediate the ability of a fungus to adapt to the cold. Environmental Microbiology 12: 810–820. doi: 10.1111/j.1462-2920.2009.02127.x 20050869 10.1111/j.1462-2920.2009.02127.x

[79] Salichos L, Rokas A (2010) The diversity and evolution of circadian clock proteins in fungi. Mycologia 102: 269–278. 20361495 10.3852/09-073

[80] Cheng P, Yang Y, Heintzen C, Liu Y (2001) Coiled‐coil domain‐mediated FRQ–FRQ interaction is essential for its circadian clock function in *Neurospora*. The EMBO Journal 20: 101–108. doi: 10.1093/emboj/20.1.101 11226160 10.1093/emboj/20.1.101 PMC140186

[81] Diernfellner A, Colot HV, Dintsis O, Loros JJ, Dunlap JC, et al (2007) Long and short isoforms of *Neurospora* clock protein FRQ support temperature‐compensated circadian rhythms. FEBS letters 581: 5759–5764. doi: 10.1016/j.febslet.2007.11.043 18037381 10.1016/j.febslet.2007.11.043 PMC2704016

[82] Chaves I, Pokorny R, Byrdin M, Hoang N, Ritz T, et al (2011) The cryptochromes: blue light photoreceptors in plants and animals. Annual Review of Plant Biology 62: 335–364. doi: 10.1146/annurev-arplant-042110-103759 21526969 10.1146/annurev-arplant-042110-103759

[83] Branco S, Gladieux P, Ellison CE, Kuo A, LaButti K, et al (2015) Genetic isolation between two recently diverged populations of a symbiotic fungus. Molecular Ecology 24: 2747–2758. doi: 10.1111/mec.13132 25728665 10.1111/mec.13132

[84] Connolly LR, Smith KM, Freitag M (2013) The *Fusarium graminearum* histone H3 K27 methyltransferase KMT6 regulates development and expression of secondary metabolite gene clusters. PLoS Genet 9: e1003916 doi: 10.1371/journal.pgen.1003916 24204317 10.1371/journal.pgen.1003916 PMC3814326

[85] Son H, Park AR, Lim JY, Shin C, Lee Y-W (2017) Genome-wide exonic small interference RNA-mediated gene silencing regulates sexual reproduction in the homothallic fungus *Fusarium graminearum*. PLoS Genetics 13: e1006595 doi: 10.1371/journal.pgen.1006595 28146558 10.1371/journal.pgen.1006595 PMC5310905

[86] O'Donnell K, Cigelnik E, Nirenberg HI (1998) Molecular systematics and phylogeography of the *Gibberella fujikuroi* species complex. Mycologia 90: 465–493.

[87] Stanke M, Keller O, Gunduz I, Hayes A, Waack S, et al (2006) AUGUSTUS: *ab initio* prediction of alternative transcripts. Nucleic Acids Research 34: W435–W439. doi: 10.1093/nar/gkl200 16845043 10.1093/nar/gkl200 PMC1538822

[88] Stanke M (2016) Augustus references.

[89] DePristo MA, Banks E, Poplin R, Garimella KV, Maguire JR, et al (2011) A framework for variation discovery and genotyping using next-generation DNA sequencing data. Nat Genet 43: 491–498. doi: 10.1038/ng.806 21478889 10.1038/ng.806 PMC3083463

[90] Van der Auwera GA, Carneiro MO, Hartl C, Poplin R, del Angel G, et al (2013) From FastQ data to high‐confidence variant calls: the genome analysis toolkit best practices pipeline. Current Protocols in Bioinformatics: 11.10. 11–11.10. 33. 10.1002/0471250953.bi1110s43 PMC4243306 25431634

[91] Arnold B, Corbett‐Detig R, Hartl D, Bomblies K (2013) RADseq underestimates diversity and introduces genealogical biases due to nonrandom haplotype sampling. Molecular Ecology 22: 3179–3190. doi: 10.1111/mec.12276 23551379 10.1111/mec.12276

[92] Pritchard JK, Stephens M, Donnelly P (2000) Inference of population structure using multilocus genotype data. Genetics 155: 945–959. 10835412 10.1093/genetics/155.2.945 PMC1461096

[93] Falush D, Stephens M, Pritchard JK (2003) Inference of population structure using multilocus genotype data: linked loci and correlated allele frequencies. Genetics 164: 1567–1587. 12930761 10.1093/genetics/164.4.1567 PMC1462648

[94] Evanno G, Regnaut S, Goudet J (2005) Detecting the number of clusters of individuals using the software STRUCTURE: a simulation study. Molecular Ecology 14: 2611–2620. doi: 10.1111/j.1365-294X.2005.02553.x 15969739 10.1111/j.1365-294X.2005.02553.x

[95] Pattengale ND, Alipour M, Bininda-Emonds OR, Moret BM, Stamatakis A. How many bootstrap replicates are necessary?; 2009 Springer pp. 184–200. 10.1089/cmb.2009.0179 20377449

[96] Stamatakis A (2014) RAxML version 8: a tool for phylogenetic analysis and post-analysis of large phylogenies. Bioinformatics 30: 1312–1313. doi: 10.1093/bioinformatics/btu033 24451623 10.1093/bioinformatics/btu033 PMC3998144

[97] Lewis PO (2001) A likelihood approach to estimating phylogeny from discrete morphological character data. Systematic Biology 50: 913–925. 12116640 10.1080/106351501753462876

[98] Huson DH, Bryant D (2005) Application of phylogenetic networks in evolutionary studies. Molecular Biology and Evolution 23: 254–267. doi: 10.1093/molbev/msj030 16221896 10.1093/molbev/msj030

[99] Pfeifer B, Wittelsbürger U, Onsins SER, Lercher MJ (2014) PopGenome: an efficient Swiss army knife for population genomic analyses in R. Molecular Biology and Evolution 31: 1929–1936. doi: 10.1093/molbev/msu136 24739305 10.1093/molbev/msu136 PMC4069620

[100] Takahata N, Nei M (1985) Gene genealogy and variance of interpopulational nucleotide differences. Genetics 110: 325–344. 4007484 10.1093/genetics/110.2.325 PMC1202567

[101] Watterson G (1975) On the number of segregating sites in genetical models without recombination. Theoretical Population Biology 7: 256–276. 1145509 10.1016/0040-5809(75)90020-9

[102] Hudson RR, Kaplan NL (1985) Statistical properties of the number of recombination events in the history of a sample of DNA sequences. Genetics 111: 147–164. 4029609 10.1093/genetics/111.1.147 PMC1202594

[103] Wakeley J (1996) The variance of pairwise nucleotide differences in two populations with migration. Theoretical Population Biology 49: 39–57. doi: 10.1006/tpbi.1996.0002 8813013 10.1006/tpbi.1996.0002

[104] Hudson RR, Slatkin M, Maddison W (1992) Estimation of levels of gene flow from DNA sequence data. Genetics 132: 583–589. 1427045 10.1093/genetics/132.2.583 PMC1205159

[105] SAS Institute Inc. (2012) JMP. 10.0.0 ed.

[106] Krzywinski MI, Schein JE, Birol I, Connors J, Gascoyne R, et al (2009) Circos: An information aesthetic for comparative genomics. Genome Research 19: 1639–1645. doi: 10.1101/gr.092759.109 19541911 10.1101/gr.092759.109 PMC2752132

[107] Waterhouse AM, Procter JB, Martin DM, Clamp M, Barton GJ (2009) Jalview Version 2—a multiple sequence alignment editor and analysis workbench. Bioinformatics 25: 1189–1191. doi: 10.1093/bioinformatics/btp033 19151095 10.1093/bioinformatics/btp033 PMC2672624

[108] Akey JM, Zhang G, Zhang K, Jin L, Shriver MD (2002) Interrogating a high-density SNP map for signatures of natural selection. Genome Research 12: 1805–1814. doi: 10.1101/gr.631202 12466284 10.1101/gr.631202 PMC187574

[109] Excoffier L, Hofer T, Foll M (2009) Detecting loci under selection in a hierarchically structured population. Heredity 103: 285–298. doi: 10.1038/hdy.2009.74 19623208 10.1038/hdy.2009.74

[110] Jensen JD, Thornton KR, Bustamante CD, Aquadro CF (2007) On the utility of linkage disequilibrium as a statistic for identifying targets of positive selection in nonequilibrium populations. Genetics 176: 2371–2379. doi: 10.1534/genetics.106.069450 17565955 10.1534/genetics.106.069450 PMC1950638

[111] Hohenlohe PA, Catchen J, Cresko WA (2012) Population genomic analysis of model and nonmodel organisms using sequenced RAD tags In: Pompanon F, Bonin A, editors. Data Production and Analysis in Population Genomics: Methods and Protocols. New York: Springer Science and Business Media pp. 235–260. 10.1007/978-1-61779-870-2_14 22665285

[112] O'Donnell K, Kistler HC, Tacke BK, Casper HH (2000) Gene genealogies reveal global phylogeographic structure and reproductive isolation among lineages of *Fusarium graminearum*, the fungus causing wheat scab. Proceedings of the National Academy of Sciences 97: 7905–7910. 10.1073/pnas.130193297 PMC16643 10869425

[113] Ma L-J, Van Der Does HC, Borkovich KA, Coleman JJ, Daboussi M-J, et al (2010) Comparative genomics reveals mobile pathogenicity chromosomes in *Fusarium*. Nature 464: 367–373. doi: 10.1038/nature08850 20237561 10.1038/nature08850 PMC3048781

[114] Proctor RH, Van Hove F, Susca A, Stea G, Busman M, et al (2013) Birth, death and horizontal transfer of the fumonisin biosynthetic gene cluster during the evolutionary diversification of *Fusarium*. Molecular Microbiology 90: 290–306. doi: 10.1111/mmi.12362 23937442 10.1111/mmi.12362

[115] Sieber CM, Lee W, Wong P, Münsterkötter M, Mewes H-W, et al (2014) The *Fusarium graminearum* genome reveals more secondary metabolite gene clusters and hints of horizontal gene transfer. PloS One 9: e110311 doi: 10.1371/journal.pone.0110311 25333987 10.1371/journal.pone.0110311 PMC4198257

[116] Excoffier L, Dupanloup I, Huerta-Sanchez E, Sousa VC, Foll M (2013) Robust demographic inference from genomic and SNP data. PLoS Genetics 9: e1003905 doi: 10.1371/journal.pgen.1003905 24204310 10.1371/journal.pgen.1003905 PMC3812088

[117] Gutenkunst RN, Hernandez RD, Williamson SH, Bustamante CD (2009) Inferring the joint demographic history of multiple populations from multidimensional SNP frequency data. PLoS genetics 5: e1000695 doi: 10.1371/journal.pgen.1000695 19851460 10.1371/journal.pgen.1000695 PMC2760211

[118] Hohenlohe PA, Bassham S, Etter PD, Stiffler N, Johnson EA, et al (2010) Population genomics of parallel adaptation in threespine stickleback using sequenced RAD tags. PLoS Genetics 6: e1000862 doi: 10.1371/journal.pgen.1000862 20195501 10.1371/journal.pgen.1000862 PMC2829049

[119] Quinlan AR, Hall IM (2010) BEDTools: a flexible suite of utilities for comparing genomic features. Bioinformatics 26: 841–842. doi: 10.1093/bioinformatics/btq033 20110278 10.1093/bioinformatics/btq033 PMC2832824

[120] Nguyen L-T, Schmidt HA, von Haeseler A, Minh BQ (2015) IQ-TREE: A Fast and Effective Stochastic Algorithm for Estimating Maximum-Likelihood Phylogenies. Molecular Biology and Evolution 32: 268–274. doi: 10.1093/molbev/msu300 25371430 10.1093/molbev/msu300 PMC4271533

[121] Kalyaanamoorthy S, Minh BQ, Wong TK, von Haeseler A, Jermiin LS (2017) ModelFinder: fast model selection for accurate phylogenetic estimates. Nature Methods 14: 587–589. doi: 10.1038/nmeth.4285 28481363 10.1038/nmeth.4285 PMC5453245

[122] Hoang DT, Chernomor O, von Haeseler A, Minh BQ, Le SV (2017) UFBoot2: Improving the Ultrafast Bootstrap Approximation. Molecular Biology and Evolution. 10.1093/molbev/msx281 PMC5850222 29077904

[123] Shimodaira H, Hasegawa M (1999) Multiple comparisons of log-likelihoods with applications to phylogenetic inference. Molecular Biology and Evolution 16: 1114–1116.

[124] Snipen L, Liland KH (2015) Micropan: An R-package for microbial pan-genomics. BMC Bioinformatics 16: 1 doi: 10.1186/s12859-014-0430-y 25888166 10.1186/s12859-015-0517-0 PMC4375852

[125] den Bakker HC, Switt AIM, Govoni G, Cummings CA, Ranieri ML, et al (2011) Genome sequencing reveals diversification of virulence factor content and possible host adaptation in distinct subpopulations of *Salmonella enterica*. BMC Genomics 12: 425 doi: 10.1186/1471-2164-12-425 21859443 10.1186/1471-2164-12-425 PMC3176500

[126] Kumar S, Stecher G, Tamura K (2016) MEGA7: Molecular Evolutionary Genetics Analysis version 7.0 for bigger datasets. Molecular Biology and Evolution: msw054. 10.1093/molbev/msw054 PMC8210823 27004904

[127] Conesa A, Götz S, García-Gómez JM, Terol J, Talón M, et al (2005) Blast2GO: a universal tool for annotation, visualization and analysis in functional genomics research. Bioinformatics 21: 3674–3676. doi: 10.1093/bioinformatics/bti610 16081474 10.1093/bioinformatics/bti610

[128] Götz S, García-Gómez JM, Terol J, Williams TD, Nagaraj SH, et al (2008) High-throughput functional annotation and data mining with the Blast2GO suite. Nucleic Acids Research 36: 3420–3435. doi: 10.1093/nar/gkn176 18445632 10.1093/nar/gkn176 PMC2425479

[129] Altschul SF, Gish W, Miller W, Myers EW, Lipman DJ (1990) Basic local alignment search tool. Journal of Molecular Biology 215: 403–410. doi: 10.1016/S0022-2836(05)80360-2 2231712 10.1016/S0022-2836(05)80360-2

[130] Marchler-Bauer A, Bryant SH (2004) CD-Search: protein domain annotations on the fly. Nucleic Acids Research 32: W327–331. doi: 10.1093/nar/gkh454 15215404 10.1093/nar/gkh454 PMC441592

[131] Marchler-Bauer A, Derbyshire MK, Gonzales NR, Lu S, Chitsaz F, et al (2015) CDD: NCBI's conserved domain database. Nucleic Acids Research 43: D222–226. doi: 10.1093/nar/gku1221 25414356 10.1093/nar/gku1221 PMC4383992

[132] Petersen TN, Brunak S, von Heijne G, Nielsen H (2011) SignalP 4.0: discriminating signal peptides from transmembrane regions. Nature Methods 8: 785–786. doi: 10.1038/nmeth.1701 21959131 10.1038/nmeth.1701

[133] Fang H, Knezevic B, Burnham KL, Knight JC (2016) XGR software for enhanced interpretation of genomic summary data, illustrated by application to immunological traits. Genome Medicine 8: 129 doi: 10.1186/s13073-016-0384-y 27964755 10.1186/s13073-016-0384-y PMC5154134

[134] Benjamini Y, Hochberg Y (1995) Controlling the false discovery rate: a practical and powerful approach to multiple testing. Journal of the Royal Statistical Society Series B (Methodological): 289–300.

[135] Kimura M (1980) A simple method for estimating evolutionary rates of base substitutions through comparative studies of nucleotide sequences. Journal of Molecular Evolution 16: 111–120. 7463489 10.1007/BF01731581

