# Supplementary material for: Population genomics of Fusarium graminearum reveals signatures of divergent evolution within a major cereal pathogen
Source: PLoS One. 2018 Mar 27;13(3):e0194616. doi: 10.1371/journal.pone.0194616 (PMC5870968; doi:10.1371/journal.pone.0194616)
Supplement: S1 Fig — The phylogeny was inferred using the Kimura 2-parameter model of nucleotide substitution [135] with a Gamma parameter to account for rate heterogeneity. Bootstrap values (%, based on 100 replications) ≥ 50 are indicated on branches. The tree was rooted at midpoint and drawn to scale, with branch lengths measured in the number of substitutions per site. (DOCX) [file pone.0194616.s001.docx]

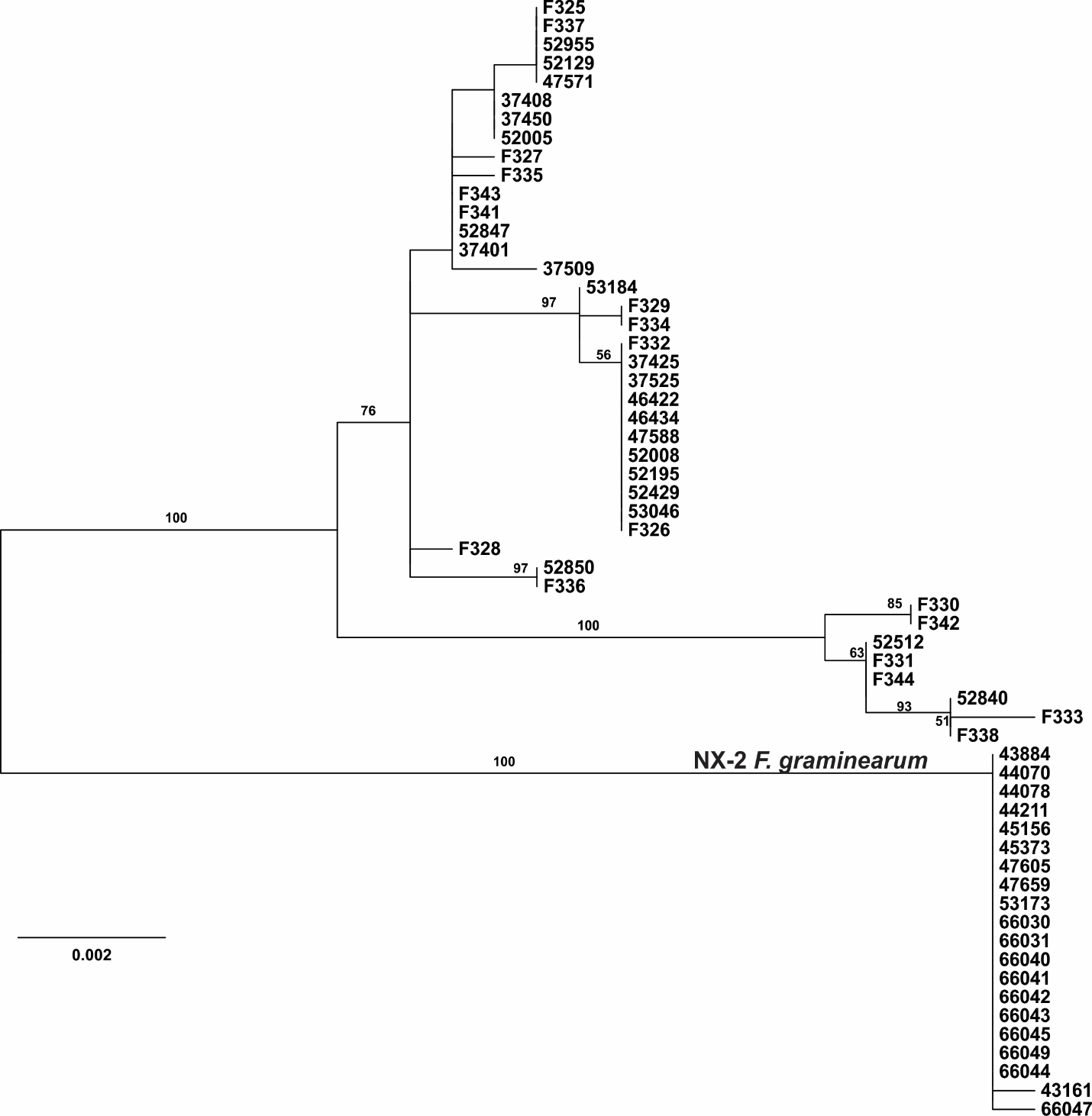
 **S1 Fig. Maximum likelihood phylogeny of *TRI1* gene sequences from the 60 isolates of *F. graminearum* used for genome sequencing***.* The phylogeny was inferred using the Kimura 2-parameter model of nucleotide substitution [135] with a Gamma parameter to account for rate heterogeneity. Bootstrap values (%, based on 100 replications) ≥ 50 are indicated on branches. The tree was rooted at midpoint and drawn to scale, with branch lengths measured in the number of substitutions per site.
