# Supplementary material for: Population genomics of Fusarium graminearum reveals signatures of divergent evolution within a major cereal pathogen
Source: PLoS One. 2018 Mar 27;13(3):e0194616. doi: 10.1371/journal.pone.0194616 (PMC5870968; doi:10.1371/journal.pone.0194616)
Supplement: S2 Fig — Maximum likelihood methods were used to construct phylogenies for 75 of the 81 genes residing in genomic regions exhibiting signatures of selection. The remaining six genes were not analyzed because homologs were not detected in one or more of the F. graminearum outgroup species (F. gerlachii, F. louisianense, F. boothii or F. pseudograminearum). Above, outlier genes located on chromosome 1, between 11.30 to 11.31 Mb are shown to exemplify discordance between phylogenies of outlier genes and the genome-wide SNP phylogeny (Fig 1). Numbers on branches indicate support values determined with 1000 ultrafast bootstraps [120,122]. The tree was rooted with F. pseudograminearum, a member of the Fusarium sambucinum species complex and a basal outgroup to the FGSC [26]. Branch lengths are drawn to scale and indicate the number of substitutions per site. (DOCX) [file pone.0194616.s002.docx]

**
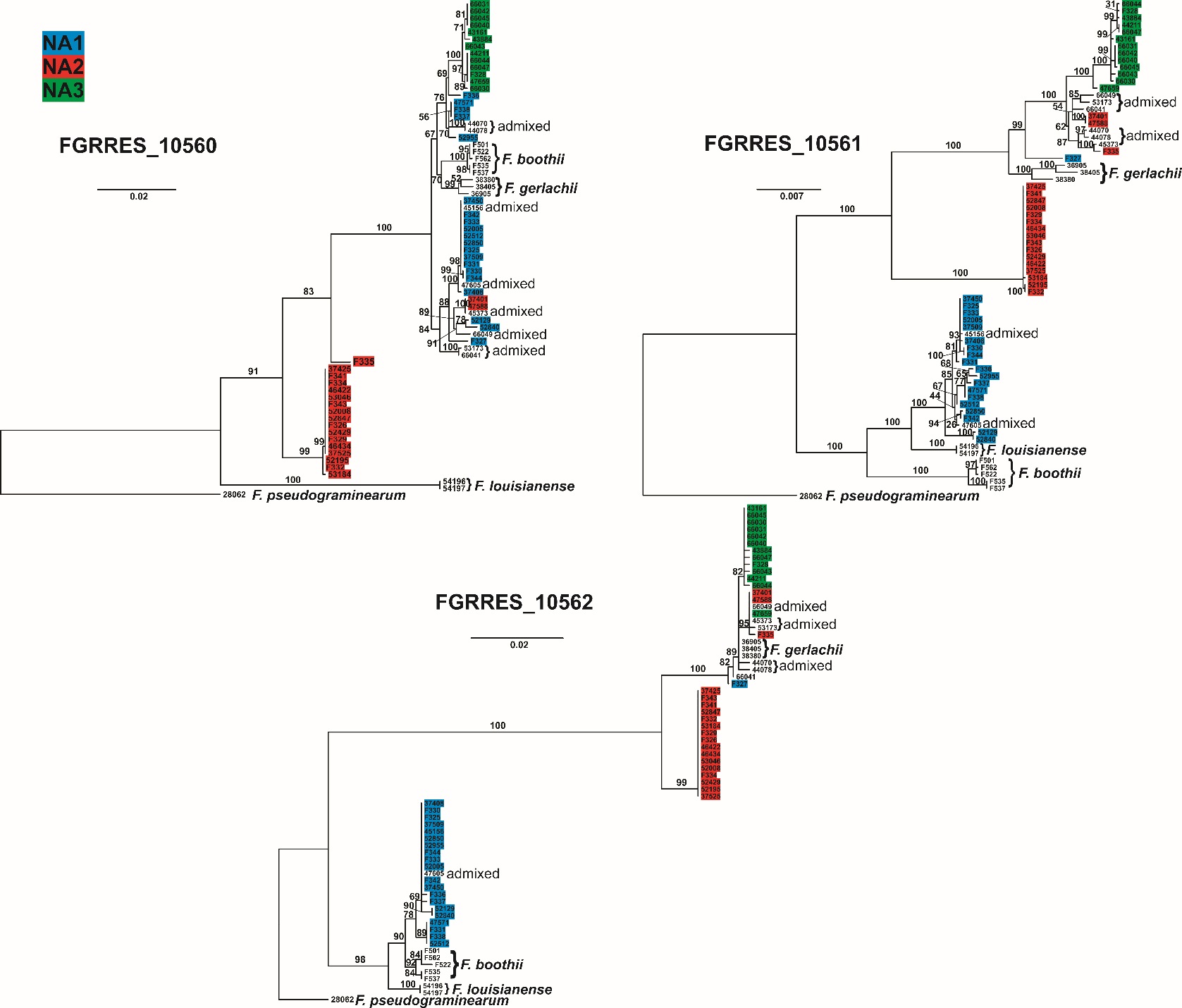
**

**S2 Fig. Outlier gene phylogenies for *F. graminearum,* *F. gerlachii, F. louisianense, F. boothii* and *F. pseudograminearum.*** Maximum likelihood methods were used to construct phylogenies for 75 of the 81 genes residing in genomic regions exhibiting signatures of selection. The remaining six genes were not analyzed because homologs were not detected in one or more of the *F. graminearum* outgroup species (*F. gerlachii, F. louisianense, F. boothii* or *F. pseudograminearum*). Above, outlier genes located on chromosome 1, between 11.30 to 11.31 Mb are shown to exemplify discordance between phylogenies of outlier genes and the genome-wide SNP phylogeny (Fig 1). Numbers on branches indicate support values determined with 1000 ultrafast bootstraps [120, 122]. The tree was rooted with *F. pseudograminearum,* a member of the *Fusarium sambucinum* species complex and a basal outgroup to the FGSC [26]. Branch lengths are drawn to scale and indicate the number of substitutions per site.
