# Supplementary material for: Population genomics of Fusarium graminearum reveals signatures of divergent evolution within a major cereal pathogen
Source: PLoS One. 2018 Mar 27;13(3):e0194616. doi: 10.1371/journal.pone.0194616 (PMC5870968; doi:10.1371/journal.pone.0194616)
Supplement: S3 Fig — Maximum likelihood methods were used to construct a phylogeny from SNPs as in Fig 1B, except sites found in outlier regions showing significant evidence of selection were omitted. All bootstrap values were 100%, except those indicated at branch nodes. The tree was rooted with F. boothii and drawn to scale, with branch lengths measured in the number of substitutions per site. (DOCX) [file pone.0194616.s003.docx]

**
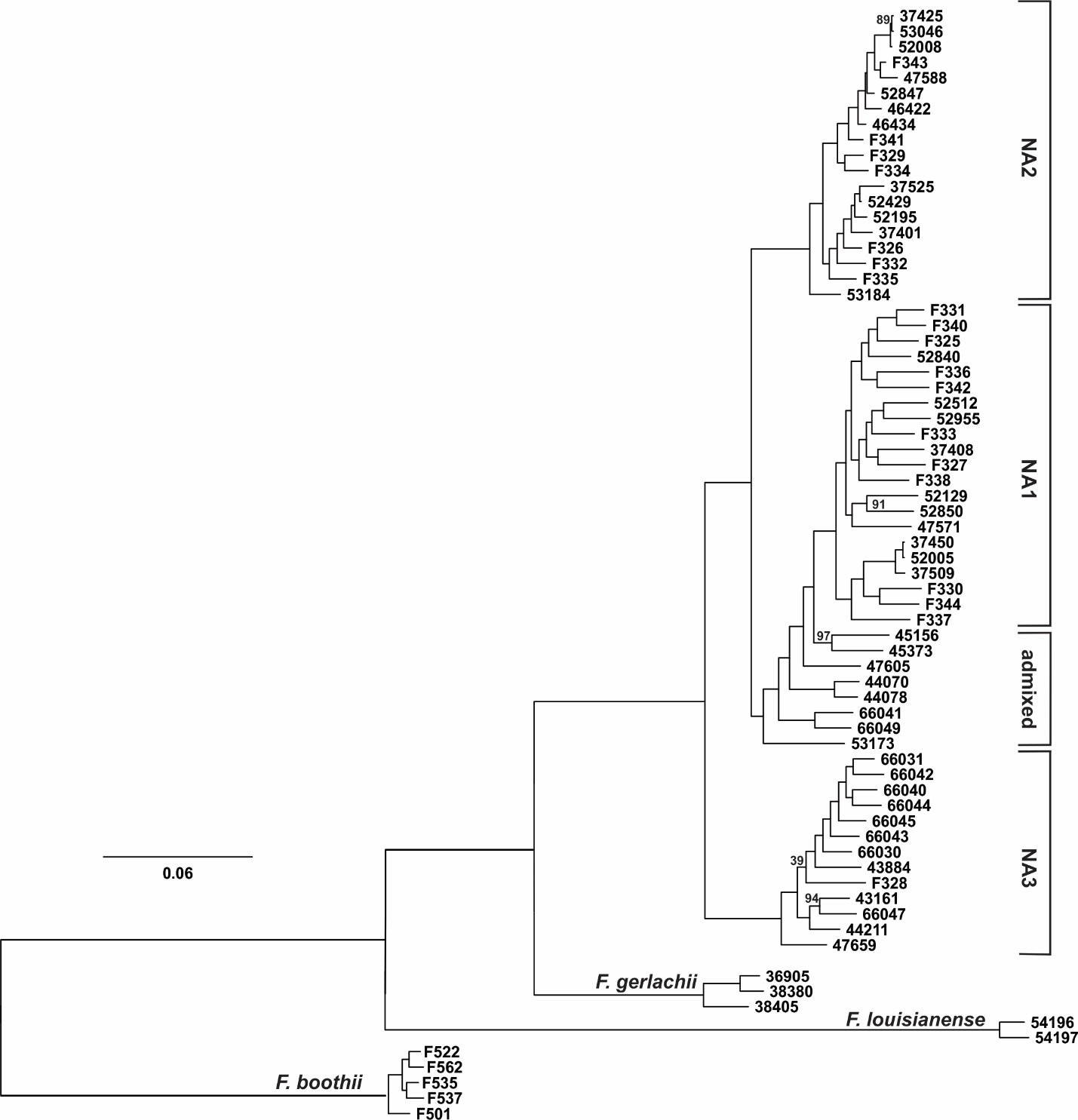
**

**S3 Fig. Genome-wide phylogeny of *F. graminearum* and related FGSC outgroup species inferred in the absence of outlier loci**. Maximum likelihood methods were used to construct a phylogeny from SNPs as in Fig 1, except sites found in outlier loci showing significant evidence of selection were omitted. All bootstrap values were 100%, except those indicated at branch nodes. The tree was rooted with *F. boothii* and drawn to scale, with branch lengths measured in the number of substitutions per site.
