# Supplementary material for: Population genomics of Fusarium graminearum reveals signatures of divergent evolution within a major cereal pathogen
Source: PLoS One. 2018 Mar 27;13(3):e0194616. doi: 10.1371/journal.pone.0194616 (PMC5870968; doi:10.1371/journal.pone.0194616)
Supplement: S4 Fig — Reference-mapped genes and orphan genes were clustered into orthologous groups based on BLAST all-versus-all comparisons of predicted protein sequences. Gene frequency was quantified based on the presence/absence of each ortholog in the 60 sampled genomes and the PH-1 reference genome. The frequency of accessory genes (orthologs found in a subset of isolates, N = 1,681) is shown above. Core genes (N = 13,632) found in all 61 genomes are not depicted in the figure. (DOCX) [file pone.0194616.s004.docx]

*
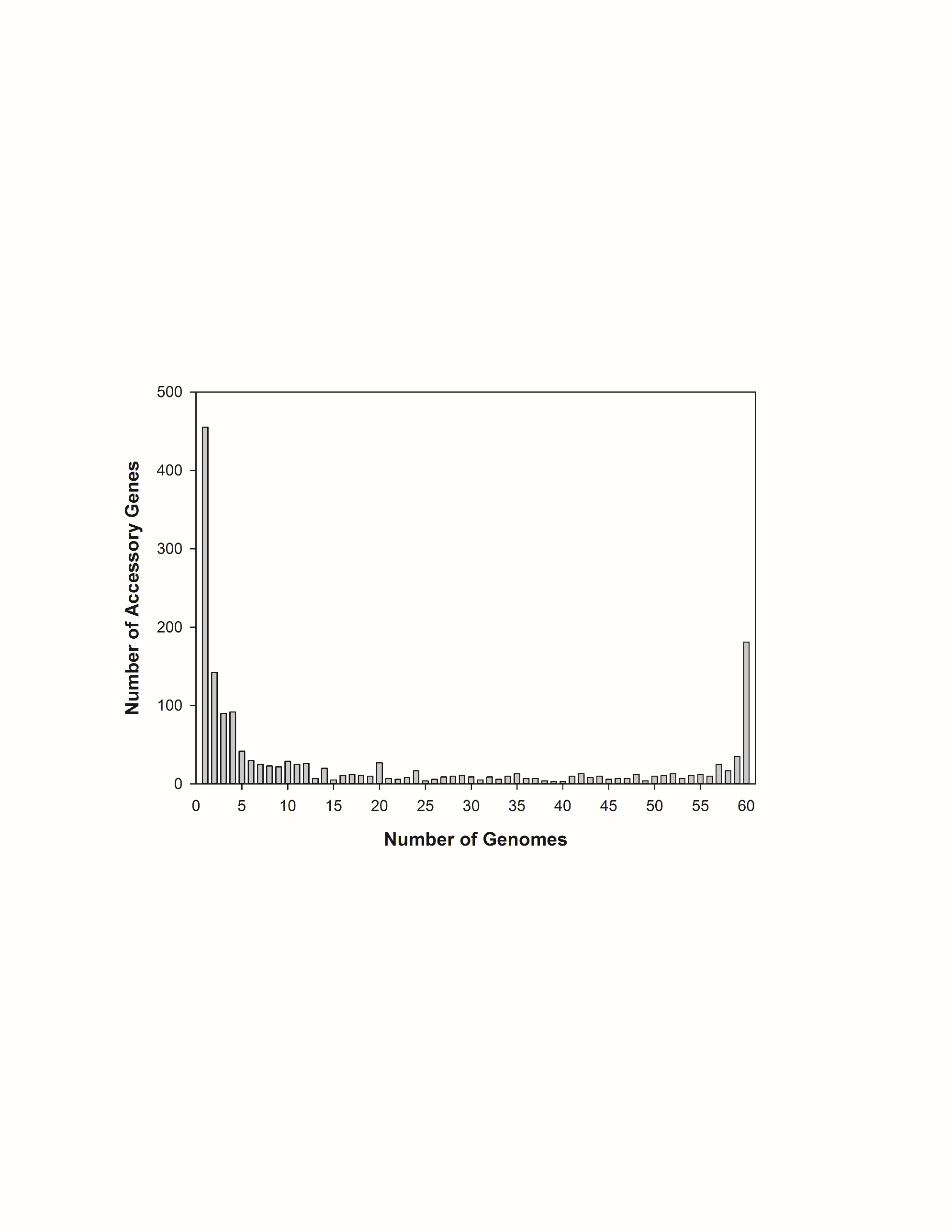
*

**S4 Fig. Frequency distribution of *F. graminearum* accessory genes.** Reference-mapped genes and orphan genes were clustered into orthologous groups based on BLAST all-versus-all comparisons of predicted protein sequences. Gene frequency was quantified based on the presence/absence of each ortholog in the 60 sampled genomes and the PH-1 reference genome. The frequency of accessory genes (orthologs found in a subset of isolates, *N =* 1,681) is shown above. Core genes (*N =* 13,632) found in all 61 genomes are not depicted in the figure.
